# A Review of Solid Electrolyte Interphases on Lithium Metal Anode

**DOI:** 10.1002/advs.201500213

**Published:** 2015-11-17

**Authors:** Xin‐Bing Cheng, Rui Zhang, Chen‐Zi Zhao, Fei Wei, Ji‐Guang Zhang, Qiang Zhang

**Affiliations:** ^1^ Beijing Key Laboratory of Green Chemical Reaction Engineering and TechnologyDepartment of Chemical EngineeringTsinghua UniversityBeijing100084P. R. China; ^2^ Joint Center for Energy Storage ResearchEnergy and Environment DirectoratePacific Northwest National LaboratoryRichlandWA99354USA

**Keywords:** solid electrolyte interphase, energy storage, lithium metal, lithium–sulfur batteries, lithium ion batteries

## Abstract

Lithium metal batteries (LMBs) are among the most promising candidates of high‐energy‐density devices for advanced energy storage. However, the growth of dendrites greatly hinders the practical applications of LMBs in portable electronics and electric vehicles. Constructing stable and efficient solid electrolyte interphase (SEI) is among the most effective strategies to inhibit the dendrite growth and thus to achieve a superior cycling performance. In this review, the mechanisms of SEI formation and models of SEI structure are briefly summarized. The analysis methods to probe the surface chemistry, surface morphology, electrochemical property, dynamic characteristics of SEI layer are emphasized. The critical factors affecting the SEI formation, such as electrolyte component, temperature, current density, are comprehensively debated. The efficient methods to modify SEI layer with the introduction of new electrolyte system and additives, ex‐situ‐formed protective layer, as well as electrode design, are summarized. Although these works afford new insights into SEI research, robust and precise routes for SEI modification with well‐designed structure, as well as understanding of the connection between structure and electrochemical performance, is still inadequate. A multidisciplinary approach is highly required to enable the formation of robust SEI for highly efficient energy storage systems.

## 1 Introduction

Rechargeable lithium (Li)‐based batteries have been world‐widely investigated as the light‐weight and high‐energy‐density energy storage devices.^1^, ^2^ The earliest commercial products of rechargeable Li batteries appeared in the 1970s, employing Li metal as the anode.^3^, ^4^ However, metallic Li electrodes were quickly discarded due to the Li dendrite growth during electrodeposition. Li dendrites can short‐circuit the cell causing a safety risk. In the 1990s, Li‐ion batteries (LIBs) were introduced by Sony Corporation to address the dendrite issues by hosting Li in a graphitic material. Li ion insertion strategies led to the tremendous success of LIBs in consumer portable electronics.^5^ It should be noted that the enhanced battery safety of LiBs is at a significant cost of energy density, because the specific capacity of Li metal (3860 mA h g^−1^) is ten times larger than that of graphite (370 mA h g^−1^). Though LIBs are now gradually approaching their theoretical limit, they still cannot meet the booming requirements for personal electronics and electric vehicles. With the development of advanced electrolytes, separators, and other battery components, Li‐metal‐based rechargeable batteries have been strongly considered in recent years.^6^, ^7^, ^8^

The safe use of Li metal as an anode is still a great challenge, as the dendritic and mossy metal deposits are very easily obtained on the working Li metal anode. Li dendrites induce a low Coulombic efficiency and severe safety risk, hindering the practical demonstration of Li metal batteries (LMBs) with very high energy density. The dendrite nucleation and growth are closely related to the surface layer between the electrolyte and anode. The surface component and structure of the layer play an extremely important effect on the morphology of Li deposits and decide the cycling performance of LMBs.^7^, ^9^, ^10^, ^11^, ^12^, ^13^, ^14^

**Figure 1:**
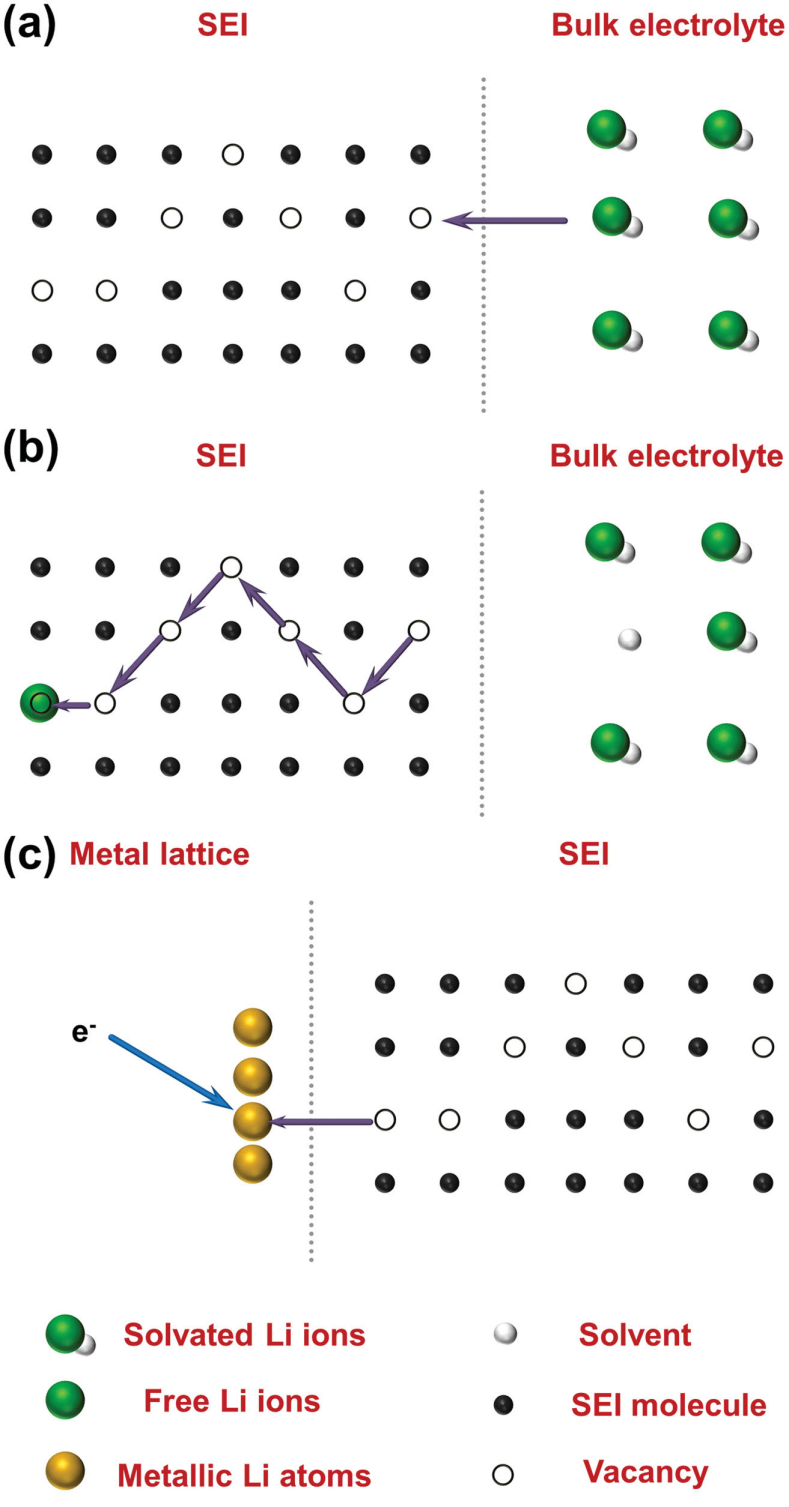
A schematic description of Li‐ion diffusion from the bulk electrolyte to the anode.^15^ a) The solvated Li ion sheds its solvent molecules and thus has the access to the internal Schottky vacancy of the SEI. b) The Li ion continuously migrates through the bulk of the SEI by relaying itself in Schottky vacancies. c) The free Li ion reaches the anode surface and accepts an electron from the current collector, then deposits as Li metal.

As Li metal can react with most organic solvents, a surface film is formed during the initial charging/discharging processes. In 1979, Peled firstly realized the electrically insulating and ionically conductive interface and named it as the solid electrolyte interphase (SEI).^15^ The SEI layer is with a thickness of ≈20 nm and includes various organic and inorganic components. On one hand, the formation of the SEI intrinsically consumes the anode and electrolyte, leading to a low efficiency. On the other hand, the SEI effectively prevents the further physical contact between Li and the solvent, therefore making Li dynamically stable in certain organic solvents. Beyond that, the SEI can adjust the distribution of Li ions from the bulk electrolyte to the anode^15^ (**Figure** **1**): (1) the solvated Li ion sheds its solvent molecules and thus has the access to the internal Schottky vacancy of the SEI; (2) the Li ion continuously migrates through the bulk of the SEI by relaying itself in Schottky vacancies; (3) finally, it reaches the anode surface and accepts an electron from the current collector, then is deposited as Li metal. The even deposition of Li ions leads to dendrite‐free morphology of LMBs with high efficiency.

The SEI film is widely observed in LIBs. In routine LIBs, robust SEI layers can be formed when graphite, silicon and other oxides are employed as the anode. Since the SEI on the graphite anode can be extremely stable during several thousands of cycles in the carbonate electrolyte, the as‐obtained LIBs with the graphite anode have been successfully commercialized. However, the SEI layer of Li metal is usually unstable and is not yet well understood. Compared with volume changes of ≈10 and 400% for graphite and silicon anodes, respectively, the relative volumetric change of Li metal anode is virtually infinite, because Li metal is ‘hostless’. The huge volume change requires the SEI layer with high elastic modulus. Dendrite growth is widely observed in LMBs, which is a fundamental challenge in LMB researche but has been detected less in current commercial LIBs. Consequently, the issue is much tougher for the SEI layer of Li metal in LMBs than that of LIBs. The fundamental exploration of SEIs on LMB is the first step to understand the complex but important issue of Li metal anode.

For an ideal SEI, it should have several features, such as high Li ionic conductivity, proper thickness with compact structure, and high elastic strength to mechanically suppress the breakthrough by Li dendrites, which is an important part to inhibit the dendrite growth on Li metal anode. However, the SEI layer of LMBs is unstable in some extreme operation conditions, such as extreme high/low temperature and high cycling rates. The formed SEI either grows much thicker or becomes non‐protective, which induces rapid performance degradation. Consequently, many strategies have been proposed to construct a stable SEI, such as the electrolyte additives, electrode design, charging models, etc.

In this contribution, the recent advances in fundamental science of the SEI formation on the Li metal anode are reviewed. Firstly, the formation mechanisms of SEI and models of SEI structure on Li metal anode are briefly summarized. The recent progress in the characterization of the components, structure, and electrochemical property of SEI layer are fully investigated. The principles of SEI formation under different operation parameters and SEI modification for robust use in a working cell are summarized. Finally, recent developments and new directions in research on SEIs of Li metal anode are also involved.

## 2 Mechanism of SEI Formation

The present electrolyte systems in LMBs mostly contain solvents and Li salts.^16^, ^17^, ^18^ The common solvents are esters (e.g. ethylene carbonate (EC), propylene carbonate (PC), dimethyl carbonate (DMC), ethylmethyl carbonate (EMC)), ethers (e.g. 1,2‐dimethoxyethane (DME), tetraethylene glycol dimethyl ether (TEGDME), 1,3‐dioxolane (DOL), tetrahydrofuran (THF)), and sulfones (dimethylsulfoxide and trimethylene sulfite). The Li salts include lithium bis(trifluoromethanesulfonyl)imide (LiTFSI), lithium bis(fluorosulfonyl)imide (LiFSI), lithium hexafluorophosphate (LiPF_6_), lithium perchlorate (LiClO_4_), and so on.

The reduction potential of organic solvent is below 1.0 V (vs. Li^+^/Li). Therefore, when bare Li is exposed to solution and a current applied, immediate reactions between Li and electrolyte species are carried out in a time constant of milliseconds or less.^19^ The insoluble products of the parasitic reactions between Li ions, anions, and solvents depositing on the metallic anode surface are regarded as the SEI.

**Figure 2:**
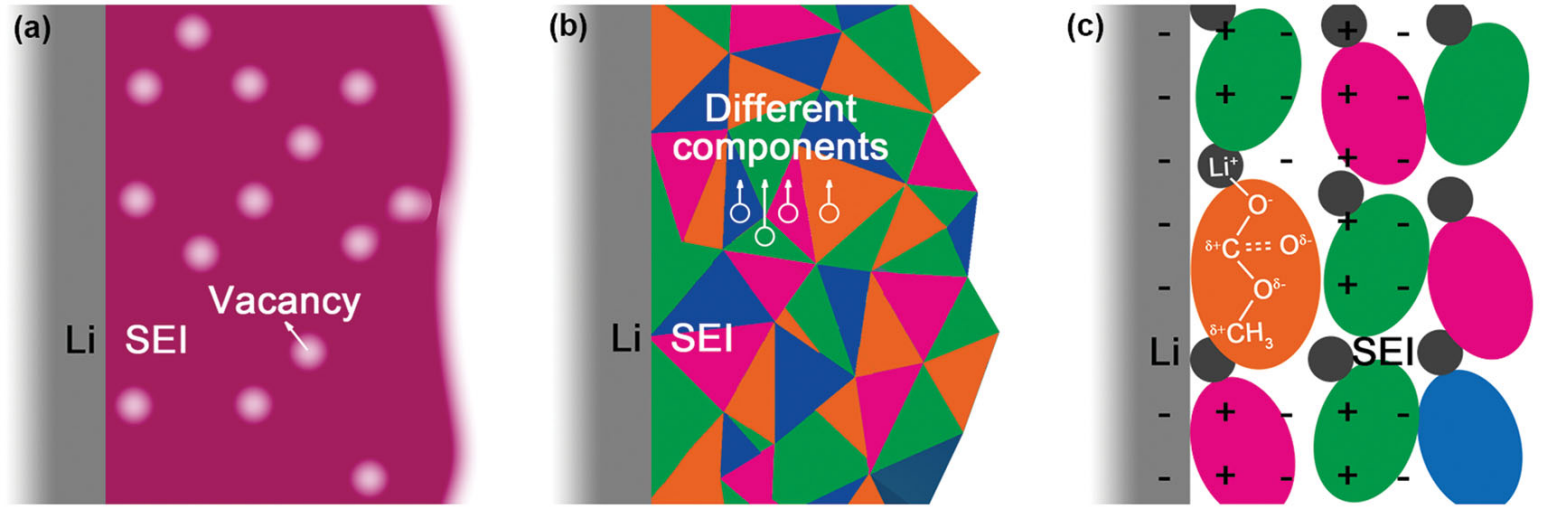
Schemes of these mechanisms for SEI formation. a) The Peled's model. Reproduced with permission.^15^ Copyright 1979, Electrochemical Society. b) Mosaic model. Reproduced with permission.^20^ Copyright 1997, Electrochemical Society. c) Coulombic interaction mechanism. Reproduced with permission.^21^ Copyright 1999, Electrochemical Society.

Though there are considerable controversies concerning the mechanism of SEI formation, several mechanisms are proposed and highlighted herein to explain SEI formation on the Li metal anode (**Figure** **2**):^15^, ^18^, ^20^, ^21^ (1) The Peled model (Figure 2a).^15^, ^22^ This is the primary mechanism to describe the SEI film established via a surface reaction. The surface reaction is stepwise and preferential reduction of certain electrolyte components. The formed layer is with integral structure except for several Schottky defects for Li ions migration. (2) The mosaic model (Figure 2b).^23^, ^24^ Relative to the preceding model, several reductive decompositions are proceeding on the negatively charged anode surface simultaneously and a mixture of insoluble multiphase products deposits on the anode. The formed SEI has a mosaic morphology, allowing the Li ions to migrate through the boundary of multiphase products. (3) The Coulombic interaction mechanism (Figure 2c).^21^, ^25^ After the surface reaction, the decomposition products are lined up with positively charged Li ion as a “head” and the partially positively charged carbons as a “foot”. The unique double electric layer allows the products to attach themselves to the existing film. Compared to the aforementioned two models, the SEI layer constructed by Coulombic interaction mechanism can have superior stability due to the stronger adhesion induced by the ion pairs.

To describe the morphology and structure of the magic layer, other structural models of SEI layers are proposed, such as (1) solid electrolyte interphase model,^15^, ^26^, ^27^ (2) polymer electrolyte interphase (PEI) model,^27^, ^28^ (3) solid polymer layer (SPL) model,^29^ (4) compact stratified layer (CSL) model.^29^

Though an impeccable model to present the real behavior of the layer between the electrolyte and anode is still lacking, the above‐mentioned models provide insightful viewpoints to understand the electrochemistry of the SEI.

## 3 SEI Characterization

### 3.1 Surface Chemistry

Both Fourier transform infrared spectroscopy (FTIR) and X‐ray photoelectron spectroscopy (XPS) are the most widely accepted technologies to probe the Li surface chemistry. Infrared spectra are collected by recording the changes in the transmitted intensity across a probed sample as a function of the wavenumber and infrared radiation can be partially absorbed at specific frequencies corresponding to their vibrational resonances. This allows for identifying functional groups by the vibrational signature of their chemical bonds.^30^, ^31^, ^32^, ^33^, ^34^, ^35^ XPS employs X‐rays of high energy (≈1200–1500 eV) to expose the sample, which allows analysis of all elements (except H and He). The elements can be accurately detected when presented in concentrations of >0.1 atomic percentage and in the outermost 10 nm of the surface.^36^, ^37^, ^38^, ^39^, ^40^ The FTIR and XPS techniques both can provide facile identification of the functional groups of organic components and types of bonds on the Li surfaces from the locations and strengths of the peaks in FTIR and XPS spectra. However, the FTIR technique is non‐destructive, while XPS may be destructive to the Li surface.^7^, ^16^, ^41^

There are significant research results that have been conducted by employing FTIR and XPS spectra.^7^, ^16^, ^17^, ^18^ The major inorganic species on Li surfaces include Li_2_O,^23^ Li_2_S/Li_2_S_2_,^42^, ^43^ LiOH, LiF,^44^, ^45^, ^46^, ^47^ LiI,^45^ Li_3_N,^42^ Li_2_CO_3_.^48^ The major organic species on Li surfaces contain ROLi, RCOOLi, ROCOLi, RCOO_2_Li, and ROCO_2_Li (R = alkyl groups). It has been reported that while organic components of the SEI are more electrochemically stable than the inorganic phases, they also significantly decrease Li transport.^49^ This in turn increases site‐specific Li deposition and induces the enhanced Li dendrite nucleation and growth as well as low efficiency. Beyond FITR and XPS, other technologies, such as Raman spectroscopy,^50^, ^51^, ^52^ Auger electron spectroscopy (AES)^53^, ^54^, ^55^, ^56^ and nuclear magnetic resonance (NMR),^57^, ^58^ are also important techniques to obtain the Li surface chemistry.

The surface chemistry of SEI layer is clearly detected by means of FTIR, XPS, Raman spectroscopy, AES, NMR etc. However, each characterization only provides limited SEI information based on its own principles. For instance, only the infrared‐active species are recorded by FTIR and the bonding with Raman scattering are collected by Raman spectroscopy, while a comprehensive analysis of all surface components can't be realized by facile FTIR/Raman spectroscopy analysis. When XPS and AES are employed for SEI characterization, the Li metal must be exposed to ultra‐high vacuum environment, which is quite different from the working Li metal anode in a battery. There are usually some overlap peaks or low signal‐to‐noise ratio in FTIR, XPS, AES, or NMR analysis. Consequently, it is a quite challenge to carry out quantitative analysis of SEI component with high precision.

**Figure 3:**
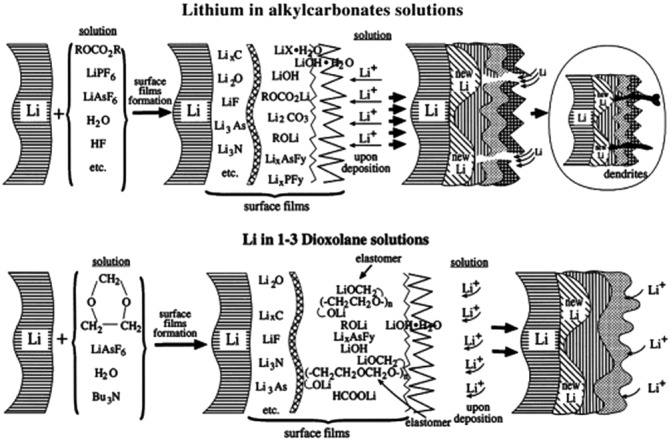
A schematic illustration of surface chemistry of SEI layer on Li electrodes in alkyl carbonates and in 1,3‐dioxolane solutions. Reproduced with permission.^16^ Copyright 2000, Elsevier.

However, the results collected by each characterization technique are complementary to each other. A full and real description of the SEI can be obtained by rational combination of these techniques. **Table** **1** lists the SEI components of Li metal anode reported in publications. The possible reactions during SEI formation are summarized in **Figure** **3**.^16^, ^41^, ^59^, ^60^, ^61^

**Table 1:** Typical contents of the SEI forming on the Li metal anode as reported in the literature

Number	Electrolyte	Component	Reference
1–1	Fresh Li plate	Outer surface: LiOH or Li_2_CO_3_, hydrocarbon, carbonate	23
		Inner part: Li_2_O, carbide	
1–2	LiBF_4_ (1.0 m) in PC for 3 days	LiF, LiOH or Li_2_CO_3_, hydrocarbon, carbonate	23
1–3	LiBF_4_ (1.0 m) in r‐BL for 3 days	LiF, LiOH or Li_2_CO_3_, hydrocarbon	23
1–4	LiBF_4_ (1.0 m) in THF for 3 days	LiF, hydrocarbon	23
2	LiC1O_4_ (1.0 m) and HF (5.0 m) in PC	Outer part: LiF, LiOH (Li_2_CO_3_ or LiOCO_2_R) Inner part: Li_2_O	24
3	LiAsF_6_ in DMC, EC‐DMC (1:1), and EC‐DEC (1:1)	ROCO_2_Li, ROLi, and Li_2_CO_3_	41
4	LiPF_6_ (1 m), vinylene carbonate (2 vol%), LiNO_3_(0.1 m), EC/DMC (1:1 Vol)	ROCO_2_Li, (‐CH_2_CH_2_O‐)*_n_*, Li_2_CO_3_, Li_3_N, LiNO_2_, LiF, C‐F	192
5–1	LiNO_3_ in DOL/DME	LiN*_x_*O*_y_*	43
5–2	Li_2_S_6_ in DOL/DME	Li_2_S, Li_2_S_2_	43
5–3	LiNO_3_ and Li_2_S_6_ in DOL/DME	Li_2_S_2_O_3_, Li_2_SO_4_, LiN*_x_*O*_y_*, Li_2_S, Li_2_S_2_	43
6	LiTFSI (0.8 m), Li_2_S_6_ (0.2 m) in DIOX/DME (1:1, v/v)	Top layer: Li_2_S_2_O_3_, Li_3_N, –NSO_2_CF_3_ Bottom layer: Li_2_S, Li_2_S_2_	42
7	LiTFSI (0.5 m), LiFSI (0.5 m) in DOL/DME (2:1, v/v)	Li_2_NSO_2_CF_3_, Li_y_C_2_F*_x_*, LiF, Li_2_S_2_O_4_, Li_2_S, etc.	47, 97

As the SEI components strongly depend on the electrode material, electrolyte salts, solvents, as well as the working state of cell, no identical SEI layer can be found in two different situations. Consequently, the actual surface chemistry of SEI layer in a given system should be obtained by in situ or even in operando characterization methods with above‐mentioned technologies rather than just referring to reported publications.

### 3.2 Surface Morphology

**Figure 4:**
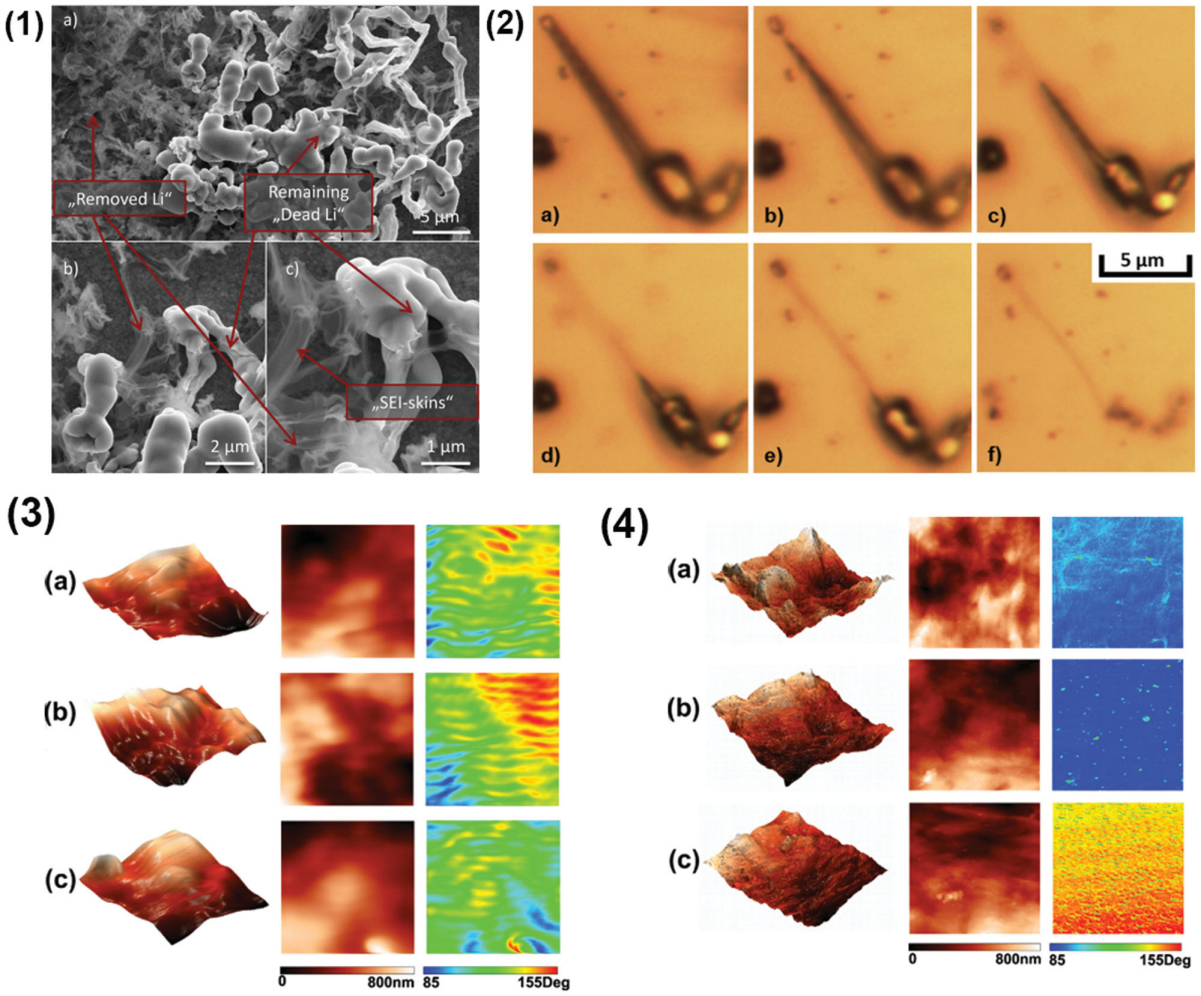
The SEI morphology on Li metal anode. 1) The appearance of Li dendrites and SEI layer on a Cu electrode after OsO_4_ exposure in constant current mode. Reproduced with permission.^63^ Copyright 2014, Elsevier. 2) In situ light microscopy of the electrodeposition of a Li needle and SEI layer after Li dissolution. Image (a) shows the initial needle and images (b–f) its dissolution. Images taken after b) 240 s, c) 285 s, d) afer 360 s, e) 390 s, and f) 585 s. Reproduced with permission.^64^ Copyright 2014, Elsevier. 3) SPM 10 × 10 μm images of Li metal immersed in a) 1.0 M LiTFSI/DME, b) 1.0 M LiTFSI/DG and c) 1.0 M LiTFSI/TEGDME for 1.0 h. From left to right column: 3D height images, 2D height images, and phase images. Reproduced with permission.^73^ Copyright 2014, Elsevier. 4) SPM 10 × 10 μm images of Li electrode cycling in a) 1.0 M LiTFSI/DME, b) 1.0 M LiTFSI/DG and c) 1.0 M LiTFSI/TEGDME for 20 cycles. From left to right column: 3D height images, 2D height images, and phase images. Reproduced with permission.^73^ Copyright 2014, Elsevier.

To directly reveal the morphology of SEI in high‐resolution, the scanning electron microscope (SEM) is the most convenient and useful choice. The cross‐sections of the individual particles in the Li metal reveals the presence of SEI layer.^62^ However, it is difficult to directly observe the SEI, since the SEI always compactly coats on the surface of anode. The structure of Li metal and SEI layer may be damaged by a high electron dose during SEM observation. Therefore, a mild SEM operation parameter with a very low electron dose and an ultra‐low accelerating voltage is recommended to investigate the SEI layer. Osmium tetroxide (OsO_4_) staining was proposed by Scheiba et al. to increase material contrast of the SEI morphology, but keep the layer untouched.^63^ After reacting with OsO_4_, SEI can be clearly distinguished from the very prominent intact Li dendrites that have readily reacted with OsO_4_ (**Figure** **4**–1). Kramer employed in situ high resolution optical microscopy to provide information on the growth and electro‐dissolution of single Li filaments.^64^ In the case of the dissolution of a Li filament, the SEI was left behind only connected to the substrate by a thin wire‐like structure (Figure 4–2), consisting of metal oxides and Li salt, e.g. LiF, or other impurities that are beneficial for Li insertion. Limited by the resolution of optical microscopy, the hyperfine structure of SEI layer cannot be obtained. To further understand the SEI morphology and investigate the layer in nanometer scale, transmission electron microscopy (TEM) experiments are employed.^65^, ^66^ Tu and co‐workers^67^ employed TEM to investigate the ex situ Li_3_N film on Li metal. The interplanar crystal spacing of (001) was measured to be 3.87 Å by detection oflattice fringes in the TEM pattern.

To investigate the structure, thickness, and mechanical property of SEI, atomic force microscope (AFM) has been employed as a powerful tool in Li‐ion battery.^68^, ^69^, ^70^, ^71^ Due to the difference in mechanical properties, the tip of the AFM can differentiate between the SEI layer and Li metal (or other substrates) and determine the thickness and modulus of SEI layer. Xu and co‐workers^72^ used in situ AFM techniques in combination with ex situ XPS to explore interfaces and obtained a comprehensive exploration into the real‐time formation of this elusive SEI film due to electrolyte decomposition. AFM characterization provides the feasibility to observe the SEI layer in the atmospheric environment. However, the imaging range is narrow and the resolution of AFM image is lower compared with images collected by SEM.

In order to directly identify the thickness of the SEI layer, Yushin and co‐workers employed secondary ion mass spectrometry, XPS, and a dual‐beam focused ion beam/SEM to characterize the thickness.^62^ The identification of an image contrasted reversal that originates from solid Li being less dense than the surrounding liquid electrolyte and electrode surface allowed SEI to be identified from Li‐containing compounds. Xie and co‐workers^73^ employed a scanning probe microscopy (SPM) system to collect height and phase image of the SEI film to understand the topography and species distribution of the SEI on Li electrode (Figure 4–3,4). The height image shows that the SEI films on the Li electrodes immersed in ether‐based electrolytes had considerably smooth surface with several raised stages. In contrast, the phase image of the SEI films had some particular features that exhibit a meshwork feature. This regular pattern was attributed to the decomposition of solvents on Li electrodes. However, the height image revealed that the SEI films on Li electrodes became rough after 20 cycles. This suggested that the decomposition reaction was more active during cycling than in the immersion of Li electrodes in electrolytes before cycling.

The microscopy characterization provides direct image evidences of SEI morphology and fine structure, which is important to infer the related physics and chemistry of SEI layers. However, the Li metal sample with SEI layer should be carefully pretreated to remove the electrolyte and other contamination from Li exposure to air and moisture. The image contrast under electron microscopy may also depend on the charged states and history of sample handling and transfer. Therefore, the explanation on the image should be very careful. The in situ and in operando technology under microscopy introduces the feasibility to understand the working Li metal, which is expected to be powerful to understand the dynamic behavior of the SEI on Li metal anode.

### 3.3 Electrochemical Properties

The electrochemical related test is always a non‐destructive, convenient and powerful technique for testing and diagnosing SEI. For instance, frequency‐domain electrochemical impedance spectroscopy (EIS) provides a facile technique to determine the complex impedance in all components of a cell (electrode, electrolyte, and their interfaces) and the semicircle in the low frequency are always be applied to represent the Li ion transfer resistance through SEI.^73^, ^74^, ^75^, ^76^, ^77^, ^78^, ^79^ Thus, EIS affords a universal opportunity to distinguish the impedance from different processes occurring simultaneously at the electrode surface. The relationship of the resistance and cell working temperature were touched upon by Archer and co‐workers.^45^ Both the interfacial and bulk (related to the lower intercept of the spectra) impedances rapidly decreased with the rise of temperature. A comparative investigation of electrolyte additives through EIS analysis on symmetric cells was carried out by Dahn and co‐workers.^80^ Almost all reported additives increased the negative electrode impedance and decreased the positive electrode impedance.

There are always EIS experiments in SEI‐related publications, which have become a routine and easy characterization method. For example, the ionic conductance of SEI layer can be obtained by the EIS experiments.^81^ However, the most troublesome step of EIS experiments is the interpretation of the original spectroscopy, because few models can clearly describe and obtain a theoretical fitting between the calculated and experimental impedances. Mostly, the interpretation of EIS is empirical. The measured spectra must be converted into electrochemical signals by fitting them to the model with professional software. Typically, the models are classified as: (i) structural ones, which link measured EIS data with kinetic parameters, concentrations and diffusion coefficients of the process, and (ii) formal ones, which describe the experimental impedances to obtain a good fitting between the calculated and experimental impedances, but without clear physico‐chemical significance. Thus, EIS data analysis using basic fitting model becomes the toughest step of EIS characterization, which limits the wide and quantitative applications in SEI property and related battery research.

### 3.4 Dynamic Characteristics

**Figure 5:**
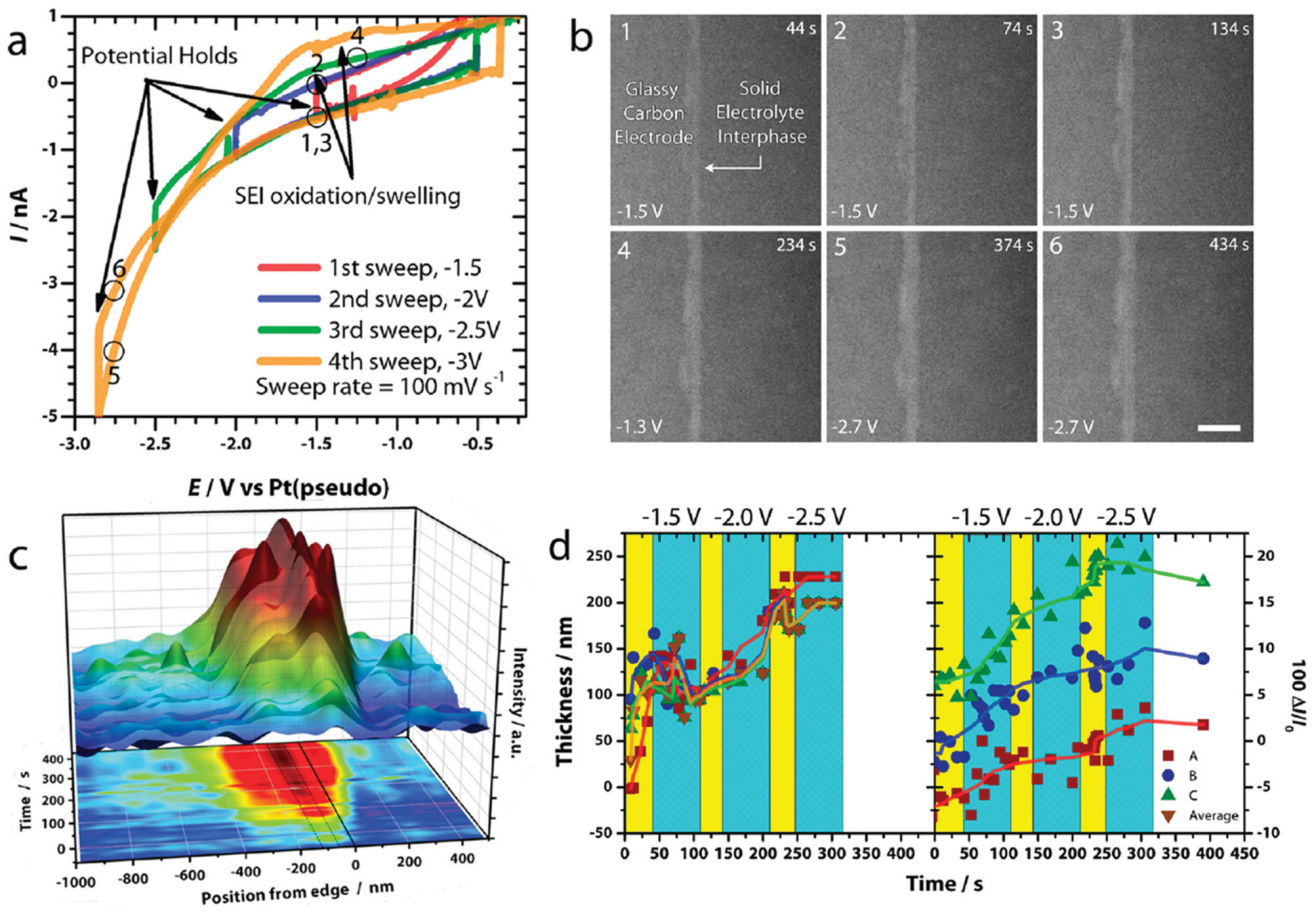
In situ ec‐S/TEM characterization of SEI.^49^ a) Sweep‐hold voltammograms of glassy carbon in 1.2 M LiPF_6_ in EC/DEC electrolyte. b) A family of STEM images exhibiting SEI evolution during the sweep‐hold experiment. The correlated Points 1–6 are described in the profile exhibited in (a). Scale bar in (b) is 1 μm. c,d) The relationship between the SEI thickness/relative image intensity and time. The colored regions in c&d relate to potential sweeps (yellow) and potential holds (cyan). Reproduced with permission.^49^ Copyright 2015, American Chemical Society.

As the SEI is continuously renewed in dynamics, it is critically important to characterize the layer in a working cell. Recent advances in micro‐fabricated electrochemical cells employing in situ and in operando technologies have opened new pathways to investigate electrochemical dynamics and to perform quantitative electrochemical measurements.^82^, ^83^, ^84^, ^85^, ^86^ These results provide comprehensive information about the morphology, structure, thickness, and mechanical property revolution of SEI layer of Li metal anode in the charging/discharging processes.^63^, ^64^, ^72^ Bieker and co‐workers^87^ conducted in situ electrochemical investigations of SEI and dendrite growth on the Li metal anode. Yang and co‐workers^33^ explored in situ micro‐FTIR spectroscopy to investigate the SEI layer between Li metal and Li/PEO_20_–LiN(CF_3_SO_2_)_2_ polymer electrolytes. The reduction reactions of oxygen and water as well as the formation of underpotential deposition Li was indicated by the cyclic voltammetric (CV) results. During the CV test, infrared spectral changes were observed and a direct correlation between the CV peaks and the magnitude of the infrared peaks can be clearly obtained. Sacci and co‐workers^49^ used in situ electrochemical scanning transmission electron microscopy (ec‐S/TEM) to perform controlled electrochemical potential sweep measurements while simultaneously imaging site‐specific structures resulting from electrochemical reactions. The SEI is approximately twice as dense as the electrolyte that determined from imaging and electron scattering theory (**Figure** **5**).

However, it should be noted that in situ and in operando technologies are always conducted in a vacuum environment with solid/ionic liquid electrolyte rather than the organic electrolyte. Most cells are always working with organic electrolyte. Consequently, when we refer the conclusion to the actual liquid system, the accuracy of the as‐obtained result may be decreased. Advanced operando characterization of the Li metal in LMBs should be further explored to collect more reliable and dynamics information of the SEI on the working Li metal anode.

### 3.5 Atomic Understanding by Theoretical Calculation

Numerous explorations have been carried out to investigate the SEI layer during the past four decades. However, it is difficult to identify and obtain the microscopic origins of working cells by experiments only, since the facility to directly observe the transient processes of the SEI formation on Li metal is too complicated. In contrast, it is very fortunate to find some theoretical studies that already afford the important insights into the SEI revolution of a working cell.^88^, ^89^, ^90^, ^91^, ^92^, ^93^

**Figure 6:**
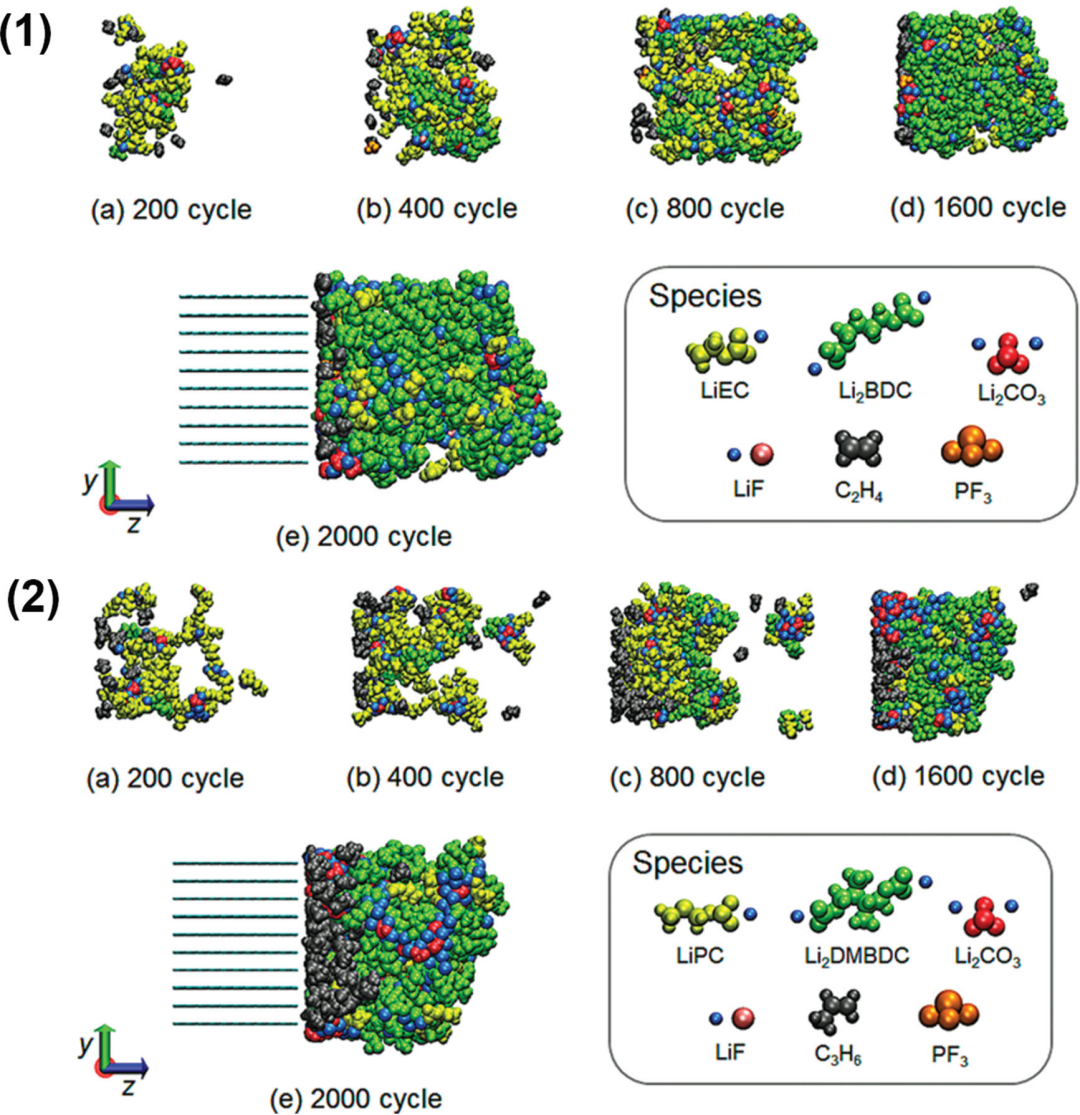
SEI layer formation illustration for different electrolyte systems.^95^ 1) EC‐based electrolyte and 2) PC‐based electrolyte: Typical snapshots of the aggregation state changes of reaction products (both bulk EC and PC not shown) in the a) 200^th^ cycle, b) 400^th^ cycle, c) 800^th^ cycle, d) 1600^th^ cycle, and e) 2000^th^ cycle. Reproduced with permission.^95^ Copyright 2014, American Chemical Society.

Using density functional theory (DFT), Qi and co‐workers^94^ probed into the SEI with Li_2_CO_3_. Below the voltage range of SEI formation, the dominant diffusion carrier in Li_2_CO_3_ was excess interstitial Li^+^, which diffused via knock‐off mechanism to maintain higher O‐coordination, rather than direct‐hopping through empty spaces in the Li_2_CO_3_ lattice. Nagaoka and co‐workers^95^ adopted the atomistic reaction simulations with the hybrid Monte Carlo/molecular dynamics reaction method to investigate the SEI stability. The results demonstrated that the SEI film formed in EC electrolyte was dense, so as to protect the electrolyte from the further reactions. In contrast, the PC‐based SEI film was sparser and thus cannot effectively protect the electrolyte from reductive decomposition. Consequently, we can conclude that the small structural difference of electrolyte molecules at the microscopic level can strongly influence the SEI film formation (**Figure** **6**). Zhang and co‐workers^96^ introduced a molecular dynamic method to explore the electrolyte and discovered that high‐rate Li plating/stripping was achieved with increased Li^+^ concentration. Quantum chemistry studies with G09 Gaussian package using G4MP2 theory and the DFT functional in conjunction with the solvation model using full solute density were adopted by Yushin and co‐workers^46^ to reveal a feasible mechanism of SEI formation. During electrolyte reduction in the LiFSI based electrolytes, the FSI(‐F) anion radicals were generated and initiated the coating formation. Such a reduction additionally renders the formation of SEI layer with LiF.

Though the theoretical calculation provides much atomic understanding of SEI layer, the calculation is mostly based on the ideal environment with large quantities of hypotheses, which is with a broad gap from the working condition of SEI layer on Li metal. Consequently, much more optimized methods should be developed to simulate the working SEI layer.

A family of analytical techniques affords detailed information on the components, morphologies, structure of the SEI layer on Li metal anode. However, none of them is capable to obtain a comprehensive understanding of the Li surface yet. FTIR, XPS, Raman spectroscopy, AES, NMR can provide the surface components of SEI layer. SEM and other high resolution microscopy can be applied to reveal the high resolution morphology of SEI. AFM is a powerful tool to investigate the structure, thickness, and mechanical property of SEI. EIS is a powerful and widely accepted technique to analyze the resistance of SEI layer in a working cell. In situ and in operando technologies are also critical to investigate morphology, structure, and electrochemical dynamics revolution of SEI layer. The theoretical chemistry is beneficial to understand the SEI at molecular scale. A rational combination of these characterization techniques is strongly considered to afford insightful understanding of the complex composition and morphology evolution of the SEI layer. More in situ and in operando methods are highly required for the dynamic characterization of SEI layer on Li metal. Since liquid electrolyte is the actual working condition of most cells, the in operando methods are expected to provide much more reliable information when they are conducted under the working conditions of batteries with organic electrolyte rather than in a vacuum environment or solid/ionic liquid electrolyte.

## 4 SEI Regulation

### 4.1 Factors Influencing SEI Formation

#### 4.1.1 Electrolyte Component

The components of SEI are always contributed from the reduction and decomposition of the electrolyte. Therefore, various electrolytes induce totally different SEI layers. Among the Li salts in Li batteries,^97^ LiClO_4_ is with strong oxidizability for Li metal that renders low safety, while LiAsF_6_ is highly toxic and LiPF_6_ exhibits poor thermostability with a few decompositions at 60–80 °C. When LiPF_6_ was employed as the Li salt, LiF would take a great proportion in the components of SEI (Equations (1) and (2)).^98^, ^99^ As the inorganic LiX is thermodynamically stable, the induced‐SEI can be protected during the cycling.

(1) $${\mathrm{LiPF}}_{6}\mathrm{(solv)}+H_{2}\mathrm{O(l)}\;\to \;\mathrm{LiF(s)}+2\mathrm{HF(solv)}+{\mathrm{POF}}_{3}\mathrm{(g)}$$

(2) $${\mathrm{LiPF}}_{6}\mathrm{(solv)}\to \mathrm{LiF(s)}+{\mathrm{PF}}_{5}\mathrm{(s)}$$

The solvents in the electrolyte must have characteristics of high conductivity, low viscosity, high flash point, and high stability. A simple, single kind of solvent cannot satisfy those demands. As a result, the solvents are always a mixture of several chemicals, such as EC and EMC, DOL and DME, etc. When EC is used as the solvent alone, the component of SEI is mostly (CH_2_OCOOLi)_2_; while when DEC/DMC is introduced, the components of SEI are mostly C_2_H_5_COOLi and Li_2_CO_3_.^48^ The uniformity of the SEI can be exceedingly enhanced by adding EC/PC to the DOL electrolytes, thus obtaining the homogeneous Li depositing and stripping.^100^

#### 4.1.2 Temperature

The SEI composition highly depended on the operation temperature. Ishiikawa and co‐workers^101^ investigated the role of temperature on SEI layer and revealed that SEI layer formed at –20 °C had compact and stable morphology and low impedance, leading to the best cycling performance. This is due to the dissolution of SEI and insertion of solvents at high temperature, and consequently poor stability of SEI. Such phenomenon was consistent with the results reported on graphite anodes.^102^ With the deepening of the research, opposite opinions were still held. The SEI layer can reform at high temperature and the dissolution and re‐depositing lead to a more compact and stable structure.^98^ Therefore, the present commercial Li‐ion cells are all pre‐treated at 30–60 °C to form the stable SEI before being released to markets. Yushin and co‐workers also found that high temperature was beneficial for the high‐energy‐density lithium–sulfur (Li–S) batteries.^103^ SEM/energy dispersive spectroscopy (EDS) investigations indicated that higher temperatures can trigger a thicker SEI on the Li anode surface to prevent polysulfide diffusion and their irreversible reduction into Li_2_S. Meanwhile, high temperature increased the inorganic components in the SEI, thus rendering a superior cycling performance with stable SEI, dendrite‐free morphology, and high efficiency.

#### 4.1.3 Current Density

During the charging process, the formation of SEI competes with Li deposition onto the current collector. The surface electrochemical reactions require additional electrons. Therefore, both the SEI morphology and structure depends on the current density. Dolle and co‐workers^104^ discovered the different steps in the passivating layer formation at a small current density with the apparition of Li_2_CO_3_ from the beginning and the appearance of ROCO_2_Li at the end of discharge. However, the SEI layer is only composed of Li_2_CO_3_ at a high current density. Kanamura and co‐workers revealed the critical role of the current density in propylene carbonate electrolyte containing LiClO_4_ and HF on SEI formation.^24^ In the Li 1s spectra, the surface of Li anode is constituted of a layer with Li_2_O (inner part) and LiF (outer part) at a current of 0.2 or 2.0 mA cm^−2^. The surface film formed on Li deposits consisted of an upper layer involving LiOH, Li_2_CO_3_, or LiOCO_2_R and a thick inner Li_2_O layer (more than 20 nm) when the current reached 10.0 mA cm^−2^. These results corresponding to the Li1s XPS spectrum, indicated that the SEI films of Li deposited at 0.2 and 2.0 mA cm^−2^ mainly consisted of LiF and the SEI film of Li deposited at 10.0 mA cm^−2^ consisted of Li_2_O.

Beyond the above‐mentioned factors (e.g. types of electrolyte including additive, temperature, and current density), other factors influencing the electrolyte, temperature, and current play a critical role on the SEI forming. When sulfur cathode is considered, there are many polysulfide intermediates in the electrolyte,^105^, ^106^, ^107^, ^108^ which definitely influence SEI layer. The structure of Li metal anode can also induce different SEI layers. On one hand, the distribution of current density and the specific current value on each anode particles depended on the Li metal shape and size.^105^, ^109^ On the other hand, the SEI forming on Li metal anode with various shapes and sizes were with different mechanical modulus.^7^ This is an important factor to inhibit Li dendrite growth and improve the Coulombic efficiency. Both the cell assembling and working pressure also affect the compactness of SEI.^110^ As a cell is a microreactor with simultaneous chemical and electrochemical reactions, consequently, the SEI layer is strongly coupled with many detailed factors. More comprehensive study of the SEI system is urgently required.

### 4.2 SEI Modification

#### 4.2.1 Electrolyte Component

**Figure 7:**
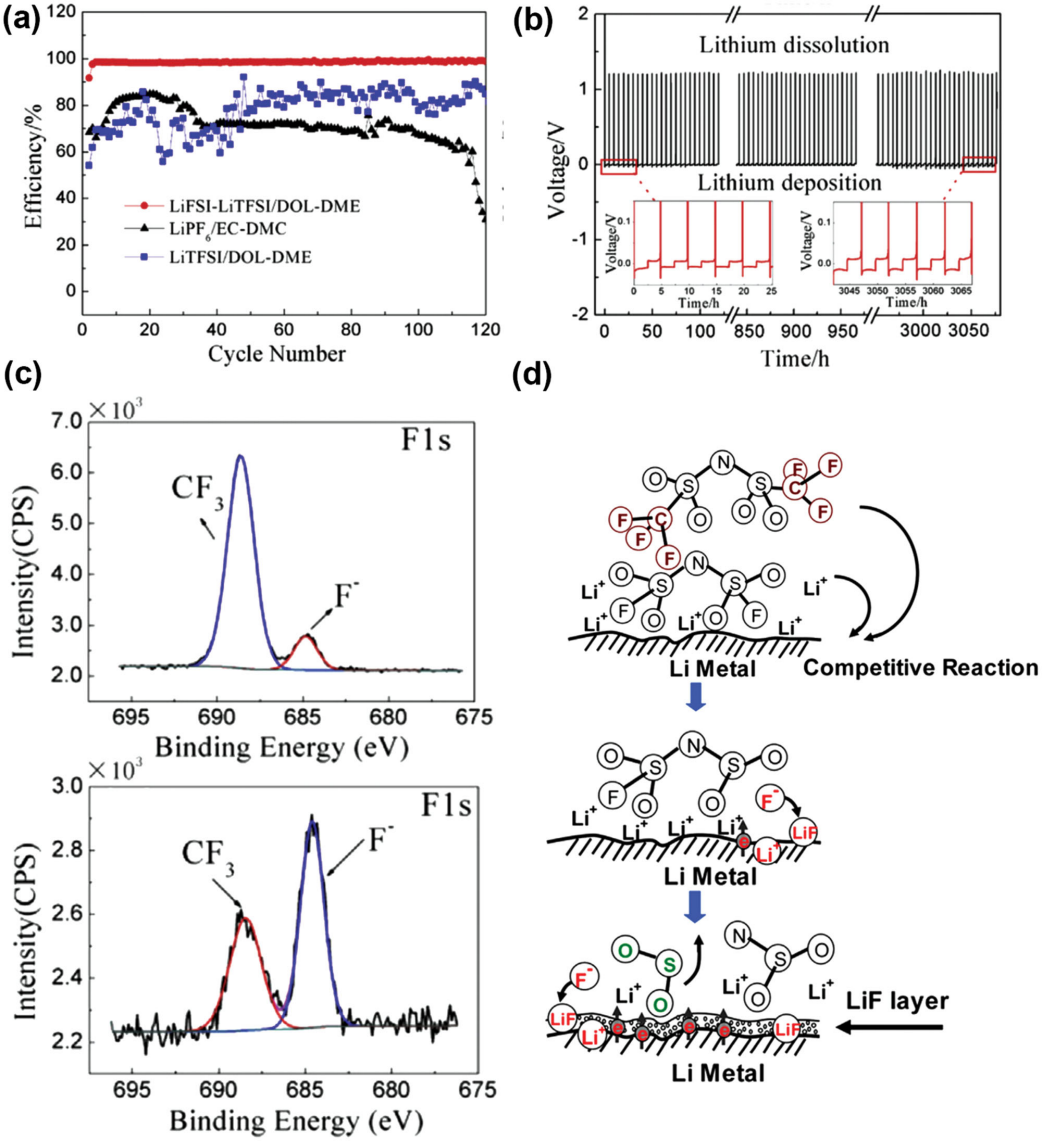
Dual‐salt electrolyte for SEI modification.^47^ a) Coulombic efficiency for different electrolyte systems. b) Galvanostatic voltage‐time curves of LiFSI‐LiTFSI/DOL‐DME electrolyte. c) XPS spectra of the F1s regions of Li surface respectively in LiFSI‐LiTFSI/DOL‐DME electrolyte (lower) and LiTFSI/DOL‐DME electrolyte (upper). d) Scheme of competitive reactions of FSI anion with Li metal at the interfaces. Reproduced with permission.^47^ Copyright 2014, Elsevier.

The cathodic and decomposing products of organic electrolyte form the dominant components of SEI. Therefore, the modulation of electrolyte is the most efficient method to stabilize SEI. As bis(fluorosulfonyl)imide (FSI^−^) anion can form a robust SEI protecting layer to prevent the liquid electrolyte from further reaction with Li metal anode, it exhibits promise as commercial electrolytes with relatively low viscosity and high chemical stability for Li‐based electric storage devices.^111^ What's more, the FSI^−^ anion can substitute the notorious TFSI^−^ to inhibit electrolytic corrosion on an Al current collector.^112^, ^113^, ^114^ Consequently, LiFSI salt dissolved in DOL/DME solvents was employed as the Li salt of electrolyte in Li metal cells that have a stable SEI and dendrite‐free morphology.^46^, ^96^, ^115^ The synergistic effect of LiFSI and DOL renders the thin and robust SEI with the homogeneous inorganic SEI inner‐layer and an elastic organic outer‐layer (e.g. poly‐DOL oligomers, CH_3_CH_2_OCH_2_OLi, HCO_2_Li, *etc*.^116^ (**Figure** **7**).^47^ The initial Columbic efficiency is 91.6% in LiFSI‐based electrolyte due to the SEI formation. The Columbic efficiency becomes stable (approximating 99%) after several cycling tests, which is significantly higher than that in LiTFSI‐based single salt and other routine electrolyte systems. Recently, Chen and co‐workers^117^ proposed the use of bis(trifluoromethanesulfonyl)‐imide/TEGDME electrolyte with LiTFSI/lithium difluoro(oxalate)borate (LiODFB) (6:4) binary lithium salts for Li–S cells. Attributed to the SEI‐forming ability of LiODFB, the Li anode was protected from suffering lithium dendrites in a working cell.

**Figure 8:**
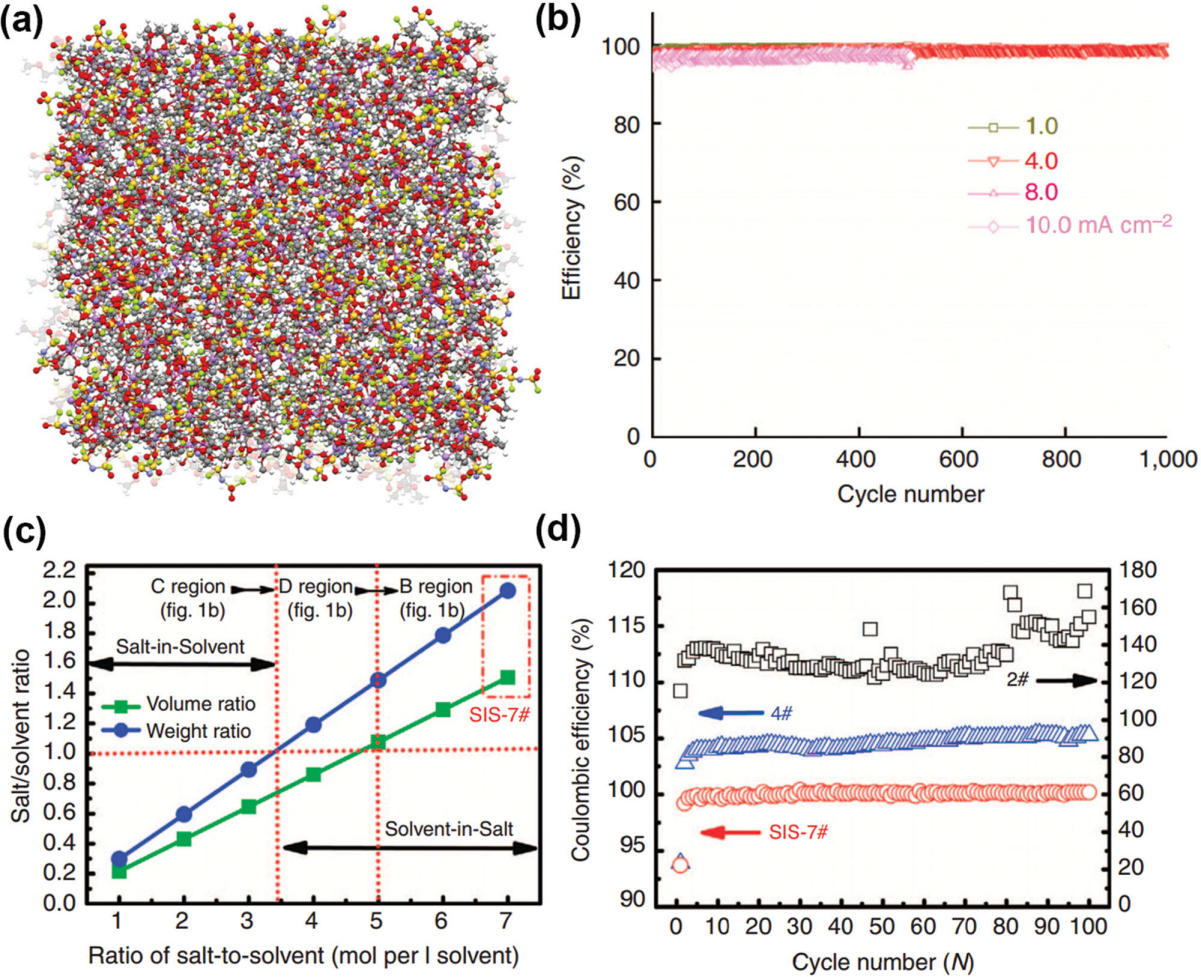
The solvent‐in‐salt electrolytes. a) Snapshots of the MD simulation boxes of 4.0 M LiFSI‐DME electrolyte. Colors for different elements: purple–Li, blue–N, red–O, green–F, and yellow–S. The uncoordinated solvent molecules (DME) are in light grey. b) Coulombic efficiency of Li deposition/striping in 4.0 M LiFSI‐DME. Reproduced with permission.^96^ Copyright 2015, Nature Publishing Group. c) Volume and weight ratio of salt‐to‐solvent with different ratios of LiTFSI to DOL:DME (volume 1:1). d) Coulombic efficiency of Li‐S cells at 0.2 C (2#: 2 mol per l solvent; 4#: 4 mol per l solvent; and SIS‐7#: 7 mol l per solvent). Reproduced with permission.^118^ Copyright 2013, Nature Publishing Group.

In the respect of solvent, a new class of ‘solvent‐in‐salt’ electrolyte has bright prospects.^118^, ^119^ In the unique electrolyte system with ultrahigh salt concentration and high Li‐ion transference number, salt holds a dominant position in the Li‐ion transport system rather than the conventional solvent. On one hand, the ultrahigh salt concentration leads to the rapid formation of a compact SEI layer to protect the anode. On the other hand, the ultrahigh salt concentration decreases the solvated Li^+^ concentration, and thus increased Li^+^ concentration renders high‐rate Li plating/stripping.^96^ A stable Coulombic efficiency of 98.4% during 1000 cycles can be achieved in this electrolyte system at a high rate of 4.0 mA cm^−2^. Even at an extremely high rate of 10.0 mA cm^−2^, a Coulombic efficiency of 97% was stably maintained for 500 cycles (**Figure** **8**a,b). The superior high‐rate performance demonstrates the very stable SEI layer. Such magic ‘solvent‐in‐salt’ electrolyte had a high Li‐ion transference number of 0.73. Thus, the cycling safety performance of next‐generation high‐energy rechargeable Li batteries is remarkably enhanced via an effective suppression of Li dendrites and shape changes in the Li metal anode. When used in a high‐energy‐density Li–S battery, the advantage of this electrolyte is further demonstrated. The ‘polysulfide shuttle phenomenon’, one of the most challenging technological hurdles of Li‐S batteries, is effectively overcome by inhibiting lithium polysulfide dissolution. Consequently, a superior long‐cycling‐stability with a Coulombic efficiency nearing 100% is achieved. (Figure 8c,d)

#### 4.2.2 Additives

**Figure 9:**
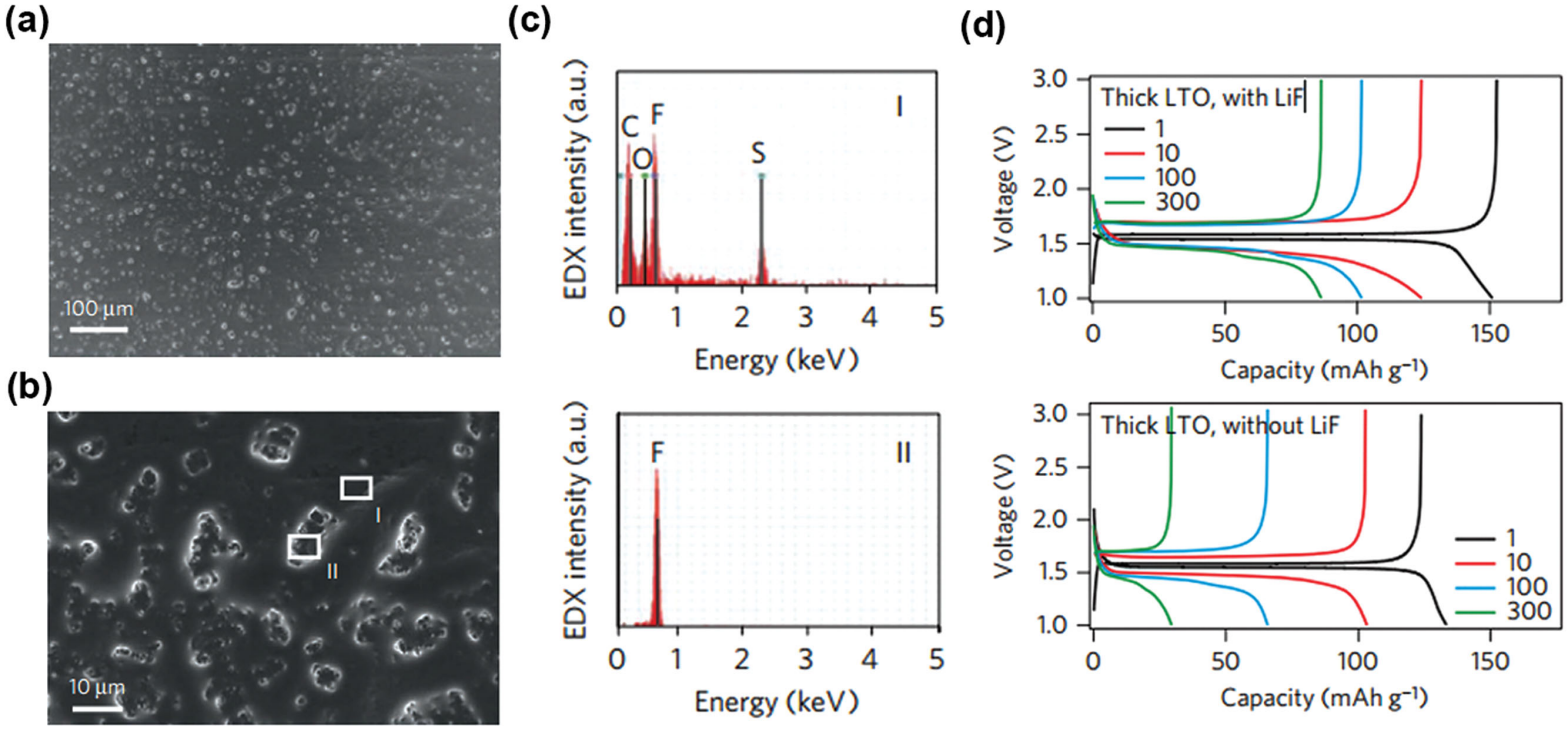
The LiF clusters on Li foil by SEM and EDS.^190^ a,b) SEM images of salt clusters on Li foils. c) EDS spectrum of region I and II exhibiting that LiF forms clusters on Li metal because of its insolubility in PC. d) The voltage vs. time profiles of thick LTO electrode with LiF and without LiF. Reproduced with permission.^190^ Copyright 2014, Nature Publishing Group.

Electrolyte additives with higher reduction voltages than solvents and salts have a positive effect on forming a stable SEI to reinforce the interfaces on the Li metal.^120^, ^121^ Archer and co‐workers employed halogenated salt blends to enhance the SEI stability and even after hundreds of charge/discharge cycles (thousands of operating hours), no signs of deposition instabilities can be observed. The EIS analysis indicated that the diffusion resistance of the Li ions through the SEI was decreased much by the halogenated salt additive.^44^, ^45^ The halogenated salt additives in the electrolyte render the SEI layer on the electrode with fluorine‐rich products (most likely LiF), LiOH and Li_2_CO_3_ driven by the in situ reactions between electrolyte and Li metal anode (**Figure** **9**). The SEI film with organic/inorganic components generates a critical effect in shedding the solvent molecules and can act as a “catalyst” to decrease the activation energy of the Li ions crossing the SEI layer and depositing on the anode.^47^, ^64^, ^111^ HF is also an important additive to produce LiF to protect the Li metal anode.^24^ PC electrolyte with 10 × 10^−3^ mol L^−1^ HF has almost the same structure as that deposited from the PC electrolyte with 5 × 10^−3^ mol L^−1^ HF and consists of both an outer LiF layer and an inner Li_2_O layer with the thickness of less than 10 nm. Manthiram and co‐workers^122^ employed fluorinated ether electrolyte to inhibit the shuttle of polysulfides and stabilize the interfaces on the Li‐metal anode without any cathode confinements or additives in a Li–S cell. The SEI layer had a composition of LiF and sulfate/sulfite/sulfide on Li metal anode, suppressing parasitic reactions between Li metal and electrolyte.

Generally, there is trace moisture in the electrolyte because of the strong water absorption of Li salts. The residual water, CO_2_, and other gases/particles presenting in non‐aqueous electrolytes has been widely regarded as a detrimental factor for Li batteries. This issue is a great problem for practical Li–air cells and these compounds even corrode the Li metal during long cycling tests.^123^ However, Osaka and co‐workers^124^ reported that the long‐cycle‐life of Li anode was twice as long and its charge transfer resistance in propylene carbonate electrolyte with saturated CO_2_ was smaller than that in the same electrolyte without CO_2_. Even with 3000 ppm H_2_O concentration in propylene carbonate electrolyte, cycle life was enhanced by CO_2_. However, such enhancement of Li cyclability with CO_2_ was effective with Ni and Ti substrates but no apparent enhancement with Cu and Ag substrates because of the catalytic reduction of CO_2_ on Cu and Ag.^125^ When the trace H_2_O (35 ppm) is accompanied by CO_2_, performance drastically improves and Coulombic efficiency reaches a maximum of 88.9%.^126^ This is attributed from the fact that trace H_2_O is found to affect the compounds of SEI on the lithium surface and produces an Li_2_CO_3_ and LiF layer on the upper part of the SEI. Very recently, Zhang and co‐workers^127^ found that a controlled trace‐amount of H_2_O (25–50 ppm) can be an effective electrolyte additive for achieving dendrite‐free Li metal deposition in LiPF_6_‐based electrolytes and avoiding detrimental effects. The trace amount of HF derived from the decomposition reaction of LiPF_6_ with H_2_O is electrochemically reduced during the initial Li deposition to get a uniform and dense LiF‐rich SEI. This LiF‐rich SEI film induces a uniform electric field on the substrate thereby enabling uniform and dendrite‐free Li deposition. CO_2_ and SO_2_ are also employed as an additive for Li electrode to increase the Li cycling efficiency.^128^, ^129^ The Li_2_CO_3_ and Li_2_SO_3_ passivation films are generated from the reaction among CO_2_, SO_2_, and Li metal, respectively. However, the toxic property of SO_2_ limits its broad applications in practical batteries. Since the beneficial effect of H_2_O, CO_2_, and SO_2_ on the robust use of Li metal was always in a very narrow concentration window, the amount of these additives should be very carefully controlled, although trace H_2_O has been widely detected in most electrolytes. The inhibition of these gas from air should be strongly considered by ration selection of permselective separator and robust electrolyte^123^ for future Li–air batteries.

**Figure 10:**
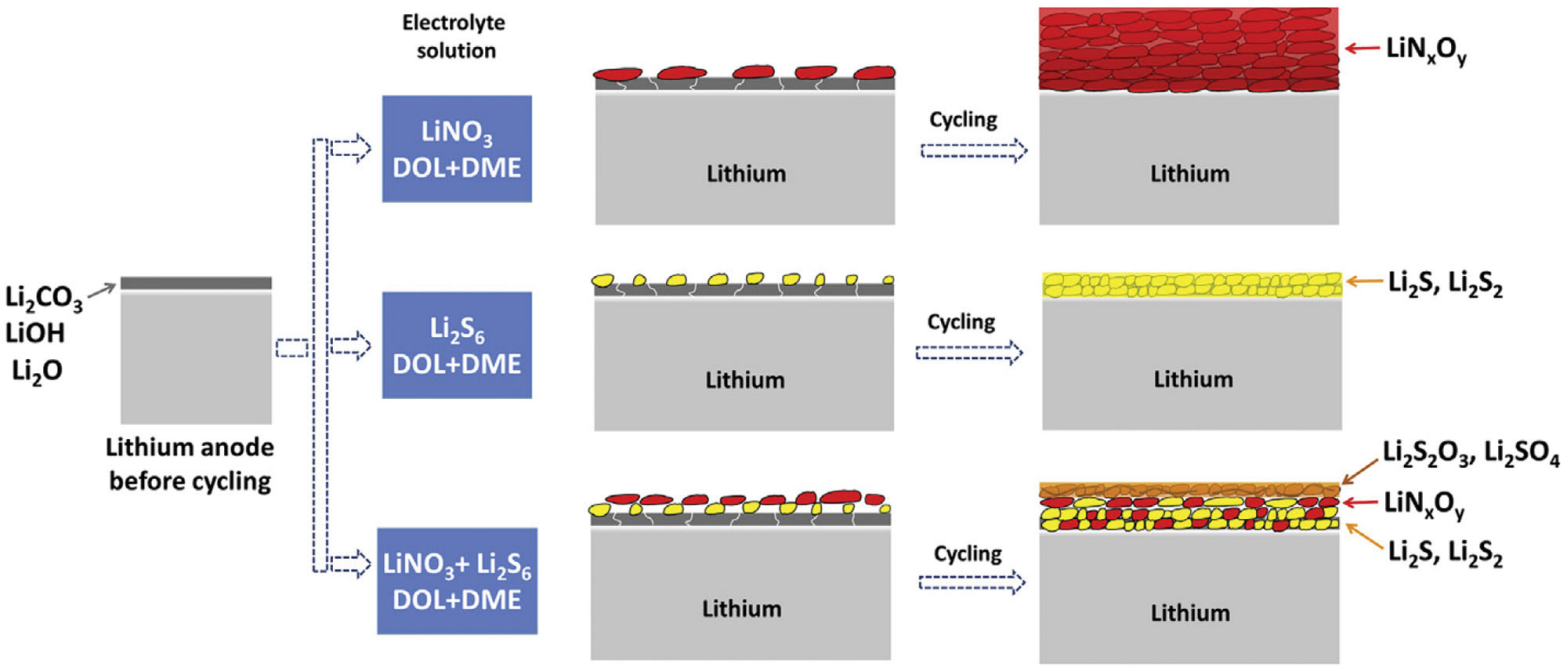
Scheme of the SEI film behavior on Li metal anode cycling in different electrolytes. Reproduced with permission.^43^ Copyright 2014, Elsevier.

Patented by Mikhaylik and co‐workers,^130^ LiNO_3_ is an important additive in the electrolyte of Li–S batteries. There is much recent progress with LiNO_3_ as electrolyte additive, utilizing LiNO_3_ in the SEI formation to protect the Li metal anode. A surface film including both inorganic species (such as LiN_x_O_y_) and organic species (such as ROLi and ROCO_2_Li) can be constructed by the strong oxidation of LiNO_3_.^59^ Based on the LiNO_3_ electrolyte, polysulfide additives may also lead to the stable SEI layer in ether electrolyte (**Figure** **10**).^43^, ^131^ Beyond LiNO_3_, other additives also exhibited superior results in improving the SEI stability and cycling performance. Due to the increased ion mobility and ionic conductivity, electrolytes with toluene additive have higher redox currents.^132^ Wang and co‐workers^133^ firstly reported BTFE with LiNO_3_ to form significantly enhanced SEI film, which is a great step to obtain stable and non‐flammable electrolyte for LMBs. Hollenkamp and co‐workers discussed the effect of LiNO_3_ additive and pyrrolidinium ionic liquid on the SEI in Li‐S cells and verified the participation of C_4_mpyr‐TFSI on the SEI formation on the anode.^134^

#### 4.2.3 ‘Artificial’ SEI Structure

**Figure 11:**
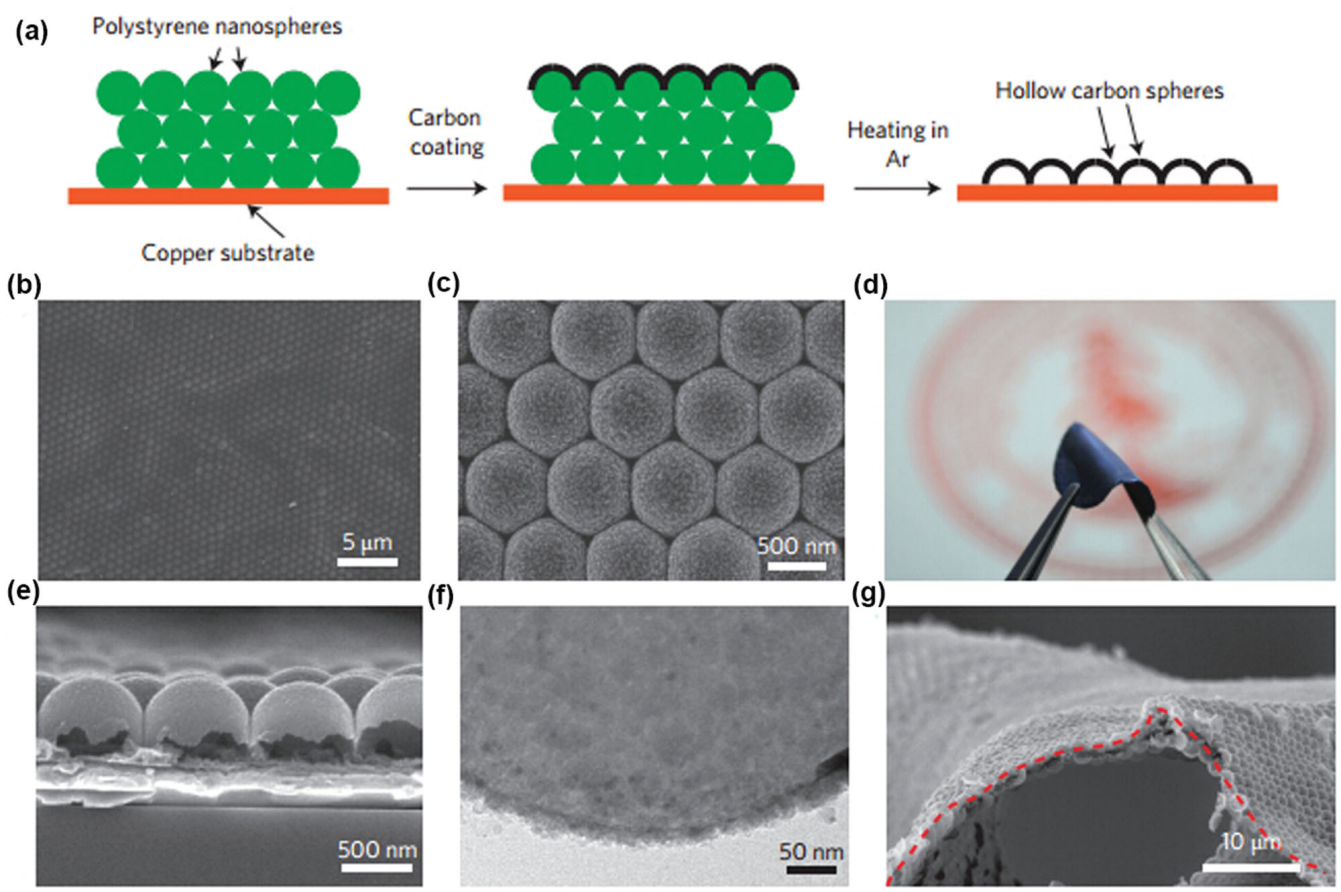
Dendrite‐free Li metal anode with hollow carbon nanospheres coating.^66^ a) Fabrication of Cu electrode with carbon nanospheres. b,c) SEM images of the carbon‐coated polystyrene nanoparticle array. d) Photo of the as‐fabricated flexible hollow carbon film after removal of the template. e) Cross‐sectional SEM and f) TEM image of the carbon nanospheres, with wall thickness of ≈20 nm. g) SEM image of the carbon nanospheres peeled off the Cu substrate. The red dashed line is the trace of the curvature of bending. Reproduced with permission.^66^ Copyright 2014, Nature Publishing Group.

**Figure 12:**
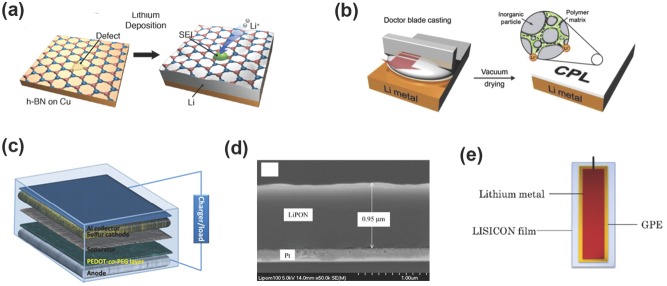
Ex‐situ‐formed protective SEI layers for Li metal anodes. a) Hexagonal boron nitride. Reproduced with permission.^71^ Copyright 2014, American Chemical Society b) Composite protective layer. Reproduced with permission.^139^ Copyright 2015, Elsevier. c) PEDOT‐co‐PEG coating layer Reproduced with permission.^138^ Copyright 2014, Royal Society of Chemistry. d) LiPON thin film. Reproduced with permission.^140^ Copyright 2004, Elsevier. e) LISICON film. Reproduced with permission.^142^ Copyright 2013, Nature Publishing Group.

The reactions among Li metal, organic solvents, Li salts, and electrolyte additives render the in situ formed SEI to protect the metallic Li anode. Another efficient approach of SEI formation is to treat the Li metal with chosen chemicals prior to its use in a cell. Thus Li electrode is covered with an ex situ formed protective layer (or ‘artificial’ SEI layer). Cui and co‐workers innovatively created an artificial SEI with a monolayer of nanostructured and interconnected amorphous hollow carbon nanospheres to protect the Li metal anode (**Figure** **11**).^66^ The artificial SEI was with a conductivity of ≈7.5 S m^−1^, effectively inhibiting the electron delivery through SEI. Large pores cannot be found on the artificial SEI, avoiding electrolyte leakage into the Li surface.^135^ When Li deposits on the surface of current collector, the hollow carbon nanosphere film is elevated, thus guaranteeing high lithiation capacity. In a Li | Cu demonstration, a high Coulombic efficiency of ≈99.5% is obtained at 0.25 mA cm^−2^ after 150 cycles. Similar to the amorphous hollow carbon nanospheres, other amorphous carbon coatings can also lead to a superior cycling stability and high Coulombic efficiency.^136^ The virtue of the artificial interfacial protection can also be provided by graphene and hexagonal boron nitride hybrid,^71^ Li_3_N/PEDOT‐co‐PEG protecting layer,^67^, ^137^, ^138^ composite protective film,^139^ ceramic coating film,^140^, ^141^ LISICON layer^142^ (**Figure** **12**).

It is acknowledged that when the shear modulus of the electrolyte is about twice that of the Li anode (≈10^9^ Pa), the dendrite suppression can be effectively achieved.^7^ Therefore, solid electrolytes with robust mechanical modulus are regarded as a multifunctional component of cell, which can not only act as the electrolyte, but also as the protective ‘artificial’ SEI layer to inhibit the dendrite growth. Though ‘artificial’ SEI layers have many advantages, the low ionic conductivity hinders their practical demonstration. Large quantities of systems are currently investigated as battery electrolytes, such as crystalline materials (oxide perovskite, La_0.5_Li_0.5_TiO_3_ 143 and thio‐LISICON, Li_3.25_Ge_0.25_P_0.75_S_4_ 144, glass ceramics (Li_7_P_3_S_11_ 145, 146, glassy materials (Li_2_S‐SiS_2_‐Li_3_PO_4_ 147, 148, polymer electrolytes^149^, ^150^, ^151^ and other materials.^152^, ^153^ Dai and co‐workers^154^ proposed a class of novel solid electrolytes with liquid‐like room‐temperature ionic conductivities (>1.0 mS cm^−1^) synthesized by taking advantage of the unique nanoarchitectures of hollow silica spheres to confine liquid electrolytes in hollow space to afford high conductivities (2.5 mS cm^−1^). Liang and co‐workers synthesized a Li_7_P_2_S_8_I phase from Li_3_PS_4_ and LiI, which exhibited the characteristics of a solid solution with electrochemical stability up to 10 V vs. Li/Li^+^ and rapid ion conduction.^155^ The unique intrinsic physical property of the solid electrolyte allows low‐temperature densification and enhanced process‐ability, which is vital to developing industrial‐scale solid electrolyte separators, as well as practical LMBs (e.g. Li–S batteries^156^).

**Figure 13:**
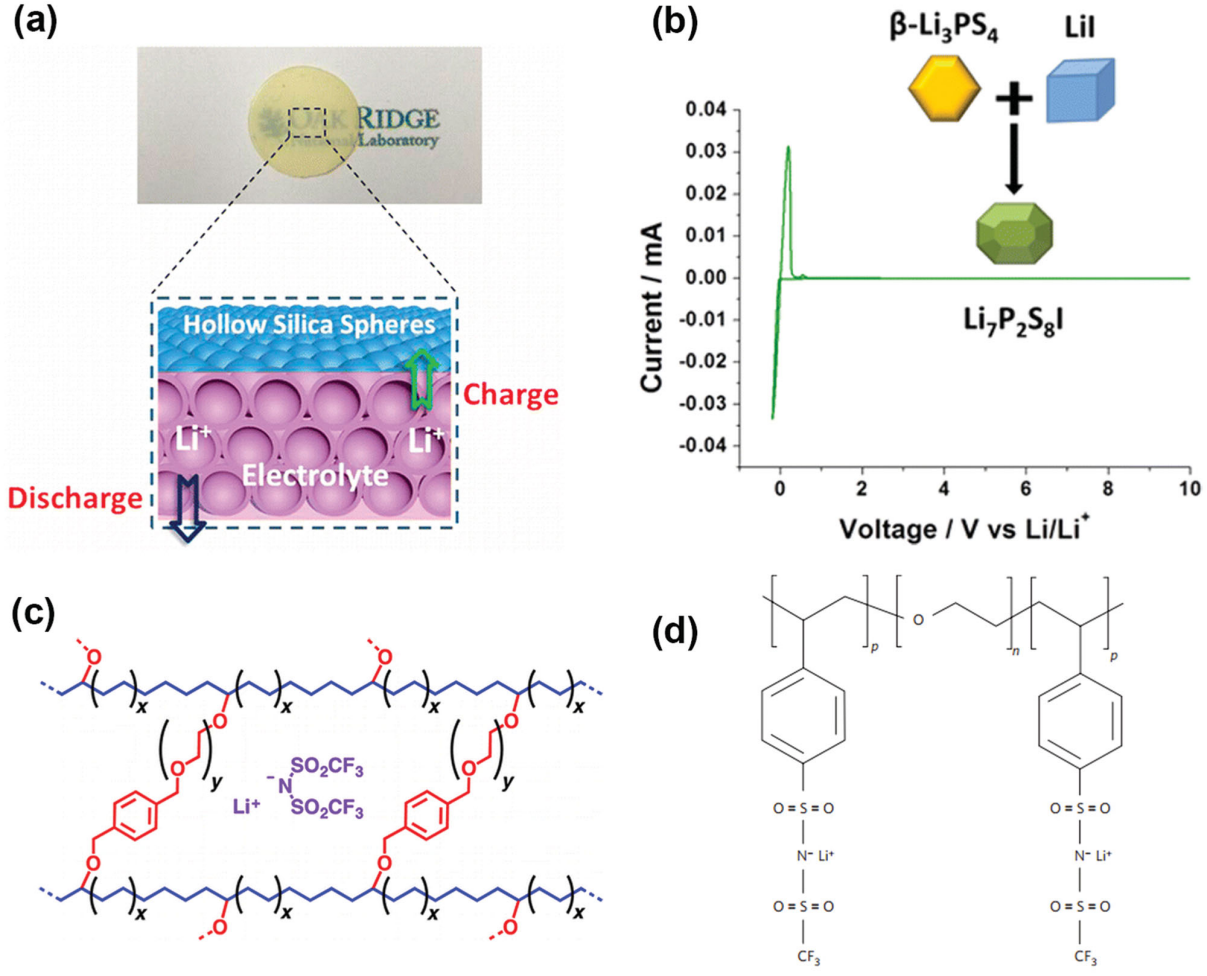
Solid electrolyte to protect the Li metal anode. a) Solid‐like electrolyte with nanoconfining liquids into the hollow structures. Reproduced with permission.^154^ Copyright 2015, Americal Chemical Society. b) Iodide‐based Li_7_P_2_S_8_I superionic conductor. Reproduced with permission.^155^ Copyright 2015, American Chemical Society. c) Cross‐linked polyethylene/poly(ethylene oxide) electrolyte. Reproduced with permission.^157^ Copyright 2014, American Chemical Society. d) Single‐ion BAB triblock copolymer. Reproduced with permission.^159^ Copyright 2013, Nature Publishing Group.

**Figure 14:**
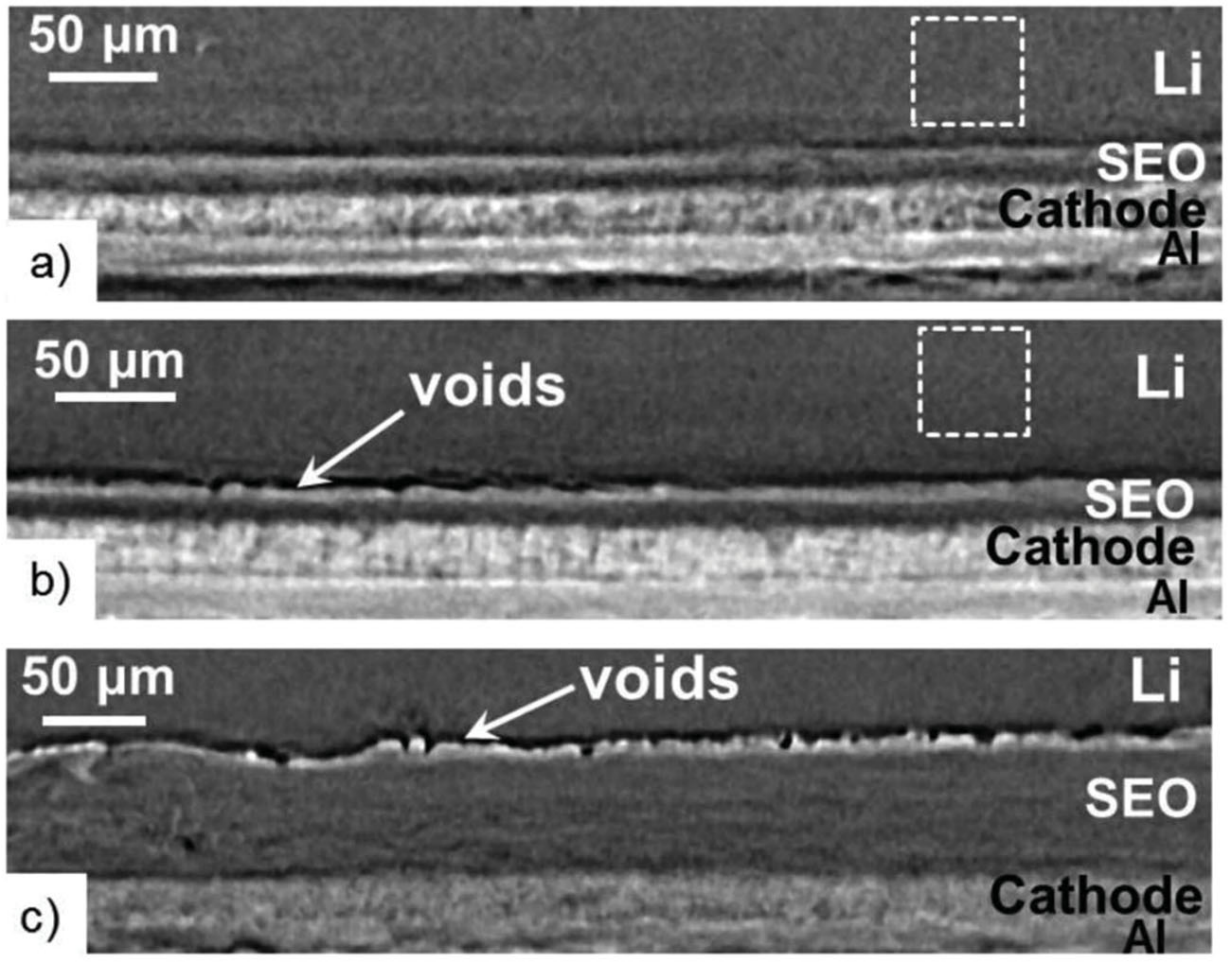
X‐ray microtomography slices exhibiting the cross‐section of Li metal polymer batteries at room temperature:^161^ a) uncycled battery annealed at 90 °C; b) battery A and c) battery B after cycling. Reproduced with permission.^161^ Copyright 2015, Electrochemical Society.

Recently, Archer's group has reported a family of pioneer research results on the solid electrolyte to improve its ionic conductivity, such as cross‐linked polyethylene/poly(ethylene oxide) electrolytes,^157^ lithiated polymer electrolyte membrane,^2^ nanoporous polymer/ceramic composite electrolytes^158^ etc., leading to increased lifetime and safety for LMBs. Single‐ion polymer^159^ and solid electrolyte^160^ are also useful strategies to improve ionic conductivity (**Figure** **13**). Besides the very low ionic conductivity, partial delamination of Li foils and the polymer/solid electrolyte layers is also responsible for capacity fade and failure.^161^ Experiments conducted by imaging the batteries using synchrotron hard X‐ray microtomography revealed that the void volume between the foil and electrolyte layer obtained after 40 to 90 cycles is comparable to volume change in the battery during one cycle (**Figure** **14**).

#### 4.2.4 Other Novel Methods

**Figure 15:**
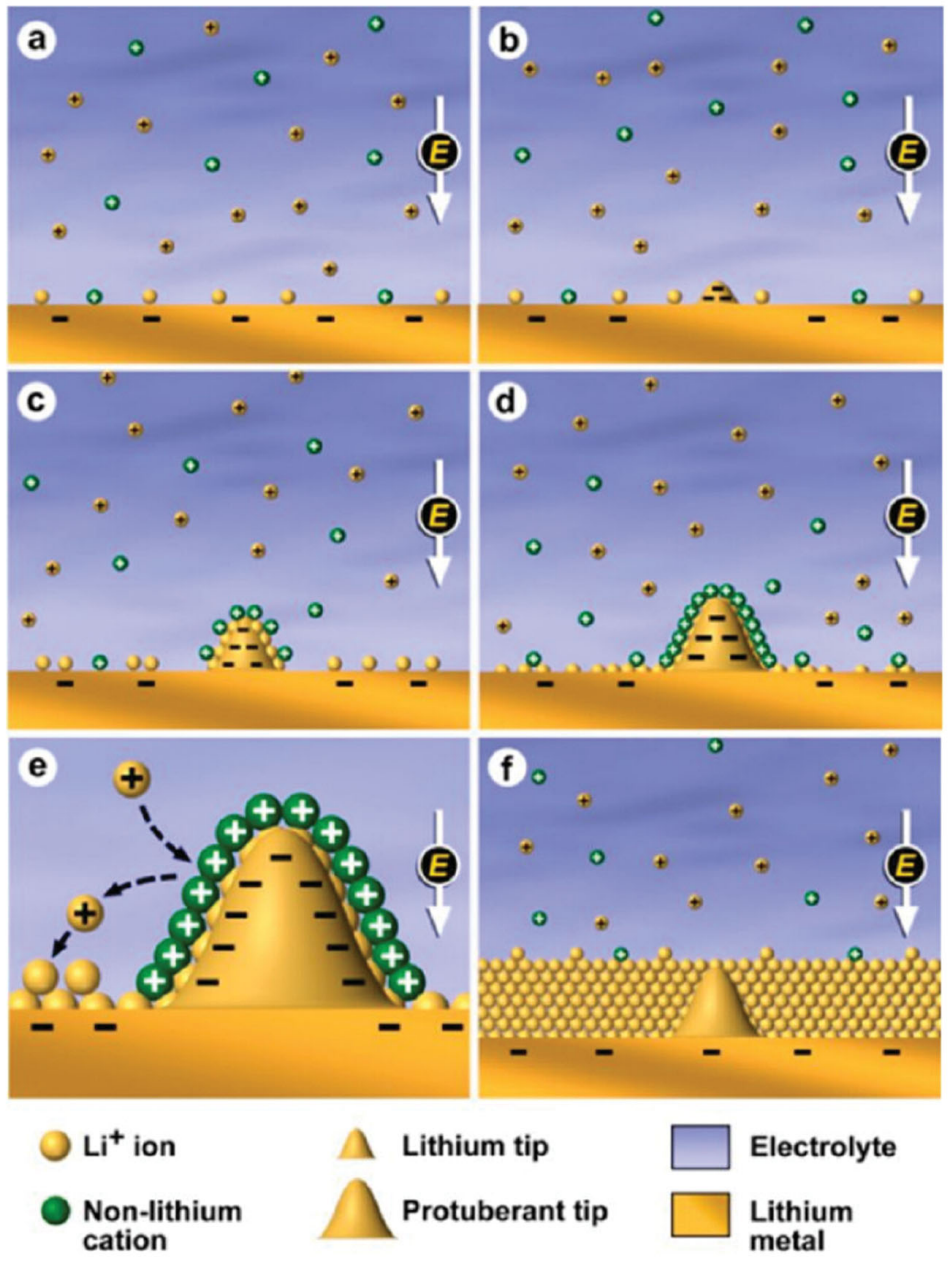
Scheme of Li deposition via the SHES mechanism. Reproduced with permission.^162^ Copyright 2013, American Chemical Society.

*Electrolyte Modification*: As the SEI is a transition layer of electrolyte and anode, any variations in electrolyte and anode will definitely exert an influence on the composites and structure of SEI. The SEI can be easily modulated to facilitate the superior cycling performance if effectively modifying the electrolyte and anode structure. Zhang and co‐workers^162^ discovered a self‐healing electrostatic shield mechanism by employing the Cs^+^ additives with proper concentration in the organic electrolyte. A positively charged electrostatic shield can be formed around the initial tip of the protuberances to force further deposition of Li to adjacent regions of the anode, thus eliminating the anisotropic dendrite formation and growth. A high Coulombic efficiency (99.86%) is associated with Li|Li_4_Ti_5_O_12_ cell, which contains excess amount of Li metal anode (**Figure** **15**). The vertically grown, self‐aligned, and highly compacted Li nanorods can be observed by modifying the concentration of Cs^+^ additives.^163^ Na^+^ also holds a similar role to Cs^+^ ions.^164^ Sodium acts to block the accelerated growth and resulting in a dimpled and dendrite‐free morphology because sodium plays a crucial role in suppressing tip‐based dendrite growth. By employing the pulse charging strategy, Hoffmann and co‐workers demonstrated that relative to direct current charging, pulse charging can significantly reduce the average dendrite length by ≈70%.^165^, ^166^ The Monte Carlo simulations dealing explicitly with Li ion diffusion, electromigration in time‐dependent electric fields were able to evidence this conclusion.

*Electrode Design*: Li foil is often used as anode material in primary and secondary Li‐metal batteries. However, the SEI forming on the Li foil is locally different in composition and morphology during cycling (corresponding to Li plating/strapping). The current density distribution is inhomogeneous because of the locally different structure of Li foil, and, in turn, the Li plating process is non‐uniform and induces a needle‐like deposition, thus leading to a low efficiency and safety risks. Some creative ideas on anode structures (e.g. Li microsphere anodes,^167^ coated Li microsphere electrodes,^109^ mechanical surface modified Li foil,^168^ graphite and Li foil hybrid anodes,^169^ Li_7_B_6_/Li framework,^170^ spatially heterogeneous carbon‐fiber papers as current collector,^4^ defect‐induced plating of Li metal within porous graphene networks,^171^ and multi‐layered graphene (MLG) coating with Cs^+^ additive in the electrolyte^172^ have been proposed and verified to retard the growth of Li dendrites.

**Figure 16:**
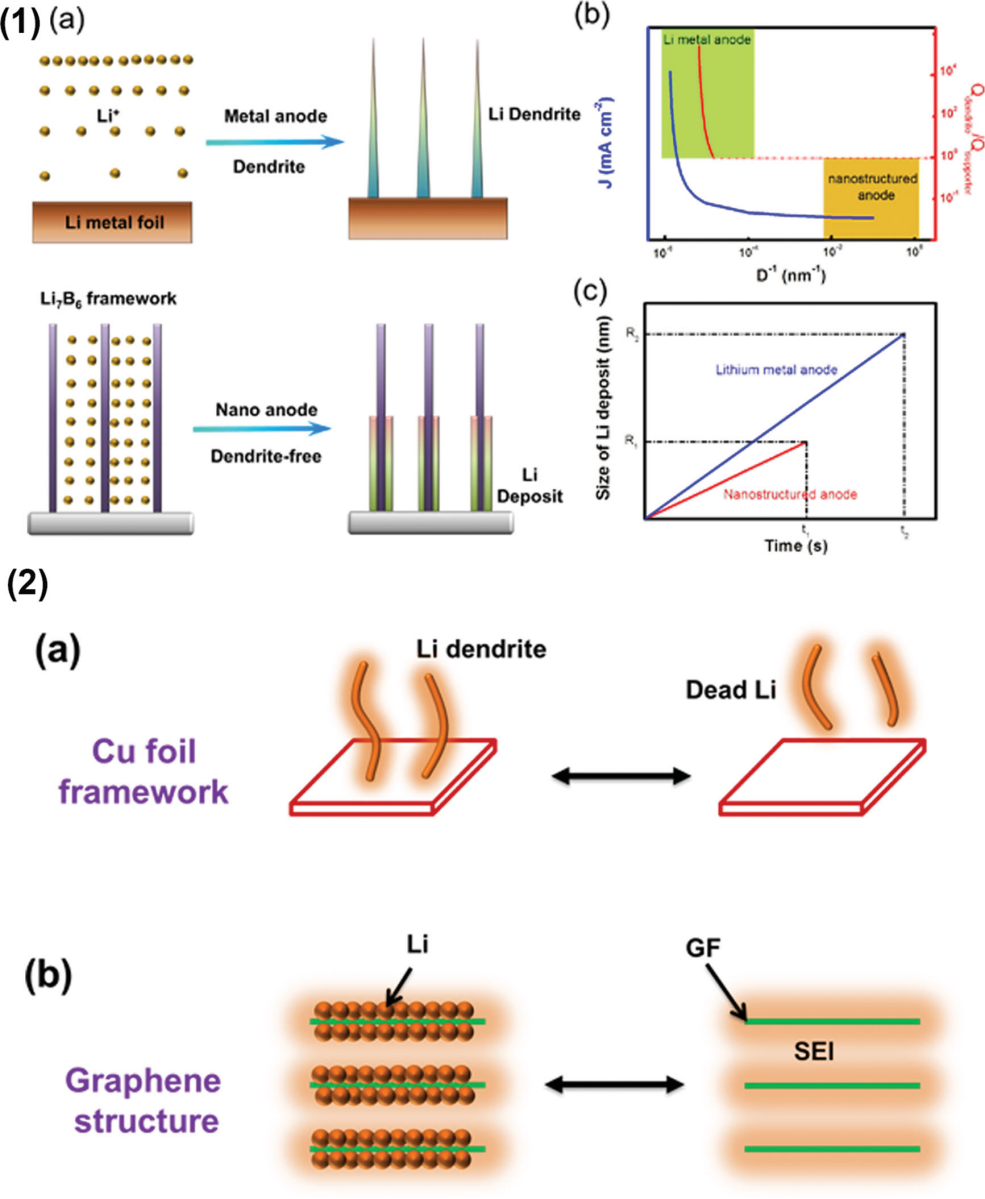
Electrode design to stabilize the Li metal anode. (1) Li depositing on the Li_7_B_6_ framework.^173^ a) Scheme of dendrite growth on the Li plate (top) and Li deposits on nanostructured anode with Li metal confined in fibrous Li_7_B_6_ matrix (bottom). b) The relationship of the current density (*J*)/relative electric field strength (*Q*_dendrite_/*Q*_supporter_) vs. matrix size. c) The dependence of the Li deposit size on the nanostructured and plate Li anode and deposit duration; R_1_, R_2_, t_1_, t_2_ were the max size of Li dendrites and the cut‐off time of dendrite‐Li on the nanostructured and plate Li metal anode, respectively (t_1_< t_2_, R_1_ < R_2_). Reproduced with permission.^173^ (2) Scheme of Li metal anode with different structures during Li deposition and dissolution.^176^ a) Without graphene framework, Li dendrites appear during Li depositing, therefore leading to much dead Li during Li dissolution. b) The SCG‐structured anode depicts a stable and uniform Li deposition and dissolution with high efficiency and low resistance. Reproduced with permission.^176^ Copyright 2015, American Chemical Society.

Zhang and co‐workers^173^ proposed a unique nanostructured anode with Li metal distributed in fibrous Li_7_B_6_ matrix (**Figure** **16**–1) as a promising anode to prevent the dendrite growth. The nanostructured anode is with a large specific area, thus rendering a low current density on the Li metal anode. The dendrite growth is effectively inhibited via decreasing the growth velocity of Li deposits and then limiting the final size of deposited Li on the nanostructured matrix, thus leading to the dendrite‐free morphology at macroscale.^11^, ^174^ The concentration gradient of Li ions near the anode surface are sharply reduced, because the 3D Li_7_B_6_ fibrous structure provides quantities of free space to accommodate electrolyte.^175^ To improve the Coulombic efficiency of Li depositing/dissolution, a dual‐phase Li metal anode containing polysulfide‐induced SEI and nanostructured graphene framework (Figure 16–2) was investigated for Li–S batteries.^176^ In a routine configuration of Li metal anode without graphene framework, Li dendrites easily grew on routine two‐dimensional (2D) substrates (such as Cu foil). As the root of dendrites can receive the electron easily and then dissolve earlier, Li dendrites easily fractured and were detached from the substrate to form dead Li, leading to the low Coulombic efficiency. If there is a pre‐existing conductive framework such as self‐supported graphene foam, the deposited Li will be well accommodated. Free‐standing graphene foam provides several promising features as underneath layer for Li anode, including (1) relative larger surface area than 2D substrates to lower the real specific surface current density and the possibility of dendrite growth, (2) interconnected framework to support and recycle dead Li, and (3) good flexibility to sustain the volume fluctuation during repeated incorporation/extraction of Li. The synergy between the LiNO_3_ and polysulfides provides the feasibility to the formation of robust SEI in an ether‐based electrolyte.^176^, ^177^ The efficient in situ formed SEI‐coated graphene structure allows stable Li metal anode with the cycling Coulombic efficiency of ≈97% with high safety and efficiency performance, which is with a low resistance of 19.65 Ω (29.10 Ω for Cu foil based Li metal anode) and high ion conductivity of 5.42 × 10^−2^ mS cm^−1^ (2.33 × 10^−2^ mS cm^−1^ for Cu foil based Li metal anode). These results indicated that interfacial engineering of nanostructured electrode were a promising strategy to handle the intrinsic problems of Li metal anodes, thus shed a new light toward LMBs, such as Li–S and Li–O_2_ batteries with high energy density.

The above‐mentioned strategies provide new insights into constructing a stable and efficient SEI to inhibit Li dendrite growth and thus to obtain a superior cycling performance of LMBs. The practical application of Li metal anode can be realized via two ways of protecting shield: (1) the nanoscale electrode that can self‐reduce the size of Li deposits to inhibit the dendrite growth, thus reinforcing the security assurance of Li metal anode; (2) a stable SEI layer that can be obtained after pre‐treating the anode in a certain electrolyte for several cycles, to protect the Li deposits from the reactions with electrolyte, therefore significantly enhancing the efficiency of Li metal anode. The synergistic effect of nanoscale electrode and stable SEI layer provides a feasible route to the commercial application of Li metal‐based batteries. However, many tiny factors, such as the assembly condition, shelving time before testing, large dead volume etc., would act significantly on the cycling performance of a coin cell.

Therefore, the rational experimental design is critically essential. To decrease the influence of cell configuration, pouch cells are an important choice with the large increase of active materials. Pouch cells are also the most approaching commercial products, thus will be the most effective demonstration to realize the proof‐of‐concept of LMBs with dendrite‐free characteristics.

## 5 Proof‐of‐Concept of LMBs

Due to safety concerns, there are not many commercial demonstrations of LMBs in portable electronics and electric vehicles, however, the huge potential of LMBs in energy storage systems motivated researchers to explore the “Holy Grail” in the lab and satisfy the demands of battery machine. Taking vehicle applications as an example, a non‐aqueous battery pack that weighs 300 kg or less will drive the vehicle if the battery can be with 140 kWh and a specific energy density of 500 Wh kg^−k^.^1^, ^178^ The 500 km range will effectively handle the “range anxiety” issue triggered by the short driving‐range. With the theoretical‐energy‐density of 3458 Wh kg^−1^ for Li metal anode, an actual‐energy‐density of 500 Wh kg^−1^ for a cell is promising to be realized when Li metal anode couples with the new‐type Li‐ion cathode (e.g. sulfur or oxygen cathode).

There are already several exciting LMB punch cell results with the optimization of the SEI layer of Li anode and the corresponding cathode and separator.^179^, ^180^, ^181^, ^182^, ^183^, ^184^, ^185^, ^186^, ^187^, ^188^, ^189^ It is critically important to note that these results are mostly achieved on the coin cells, which may be improved in a pouch cell because the ratio of active materials in a coin cell is much lower than that in a pouch cell.

**Figure 17:**
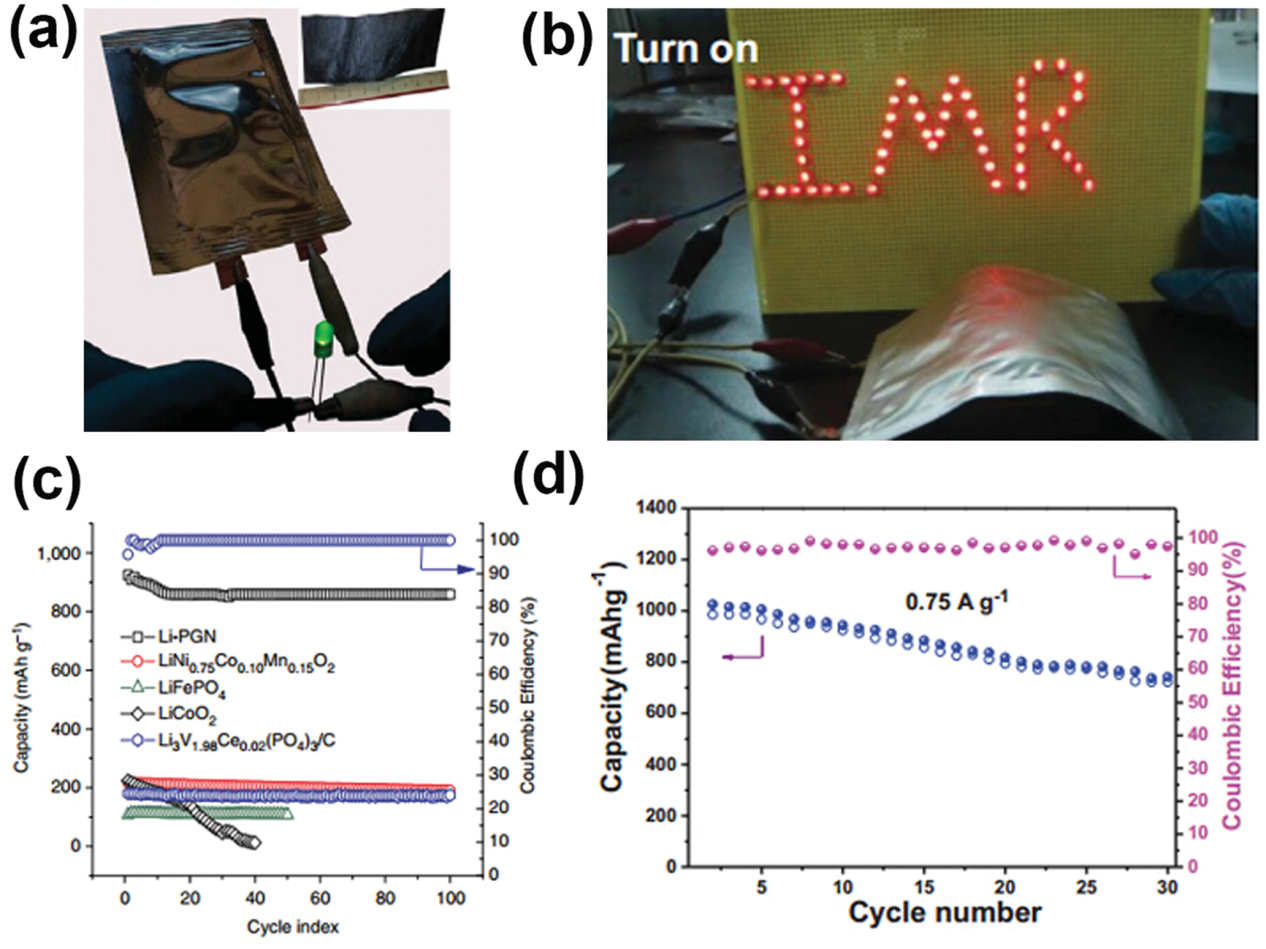
Proof‐of‐concept LMBs based on pouch cells. a) The pouch cell with full‐cell configuration of PGN anode and Li‐PGN cathode that powering an LED device (≈20 mA h). Reproduced with permission.^171^ Copright 2014, Nature Publishing Group. b) A photo of a red LED ‘IMR’ logo lighted by a bent Li–S cell. Reproduced with permission.^191^ c) Discharge capacity and Coulombic efficiency vs. cycle index of Li/PGN cathodes at a current of 1.0 C (0.30 A g^−1^). Reproduced with permission.^171^ Copright 2014, Nature Publishing Group. d) The cycling of the cell at the bent state. Reproduced with permission.^191^

To further boost the actual cell‐energy‐density of LMBs, pouch cells are constructed to improve the ratio of the active materials in the cell recently (**Figure** **17**). For a Li‐ion cell, Li metal anode affords the possibility to improve the energy density further because the cell‐energy‐density based on the intercalation anode is approaching its theoretical maximum. For the Li–S and Li–O_2_ batteries, the issue of the Li metal anode is more urgent because Li metal anodes not only provide high energy density, but also bring many troubles on the cycling performance. More and more investigations indicate that the bottleneck of LMBs lies in Li metal anode and especially the SEI layer on the anode which determines the cycling life and efficiency. A stable and efficient SEI is the basic requirement to realize broad commercial applications of LMBs.

## 6 Summary and Perspectives

Li metal is an ideal anode for rechargeable Li‐based batteries, such as Li–air, Li–S, and new types of LIBs. The formation of a stable SEI is very important to achieve high cycling efficiency and long cycling life of LMB. An ideal SEI with intrinsic minimum electrical conductivity and maximum Li^+^ conductivity should be flexible and elastic to accommodate active material breathing and non‐uniform electrochemical behavior. Remarkable progress has been achieved on the SEI components, morphology, and formation mechanism in the organic electrolyte. Effective strategies to stabilize the SEI structure through electrolyte component modulation, ‘artificial’ SEI on Li metal, nanostructured Li‐metal‐based anode, as well as solid electrolyte have been proposed and verified recently.

However, the fundamental understanding on the SEI structure, formation, property, and regulation are still inadequate. The insights on the following aspects of SEI on Li metal should be strongly considered: (1) The exact role of the SEI. Little knowledge has been gained about the role of the SEI components on the cycling performance, because the function of SEI components has not clearly been analyzed in most published literature. (2) The accurate process of Li‐ion crossing in the SEI. It is generally accepted that Li ions sheds its solvent molecules and then crosses the SEI layer before arrival at the surface of Li metal. However, such a process has not been dynamically observed and the rate is still unknown. More‐smartly‐designed in operando techniques should be developed to track the process in a working cell. (3) Controllable modification of the SEI. Many effective strategies have been proposed to modify the SEI structure. However, the modifying process is still out of control in a bulk cell because the thickness, density, ion conductivity etc. cannot yet be rationally designed.

A robust SEI layer is critical to protect the anode in a rechargeable battery for practical applications. More scientific explorations on the fundamental understanding and SEI layer building require collaborative works from physics, chemistry, materials, nanotechnology, as well as engineering communities. Through further investigation of the science and engineering of SEI on Li metal, the use of Li metal as a superior anode in a rechargeable cell is quite promising. The ultra‐stable and very robust SEI will enable broad applications of rechargeable Li metal in advanced Li–S batteries, Li–O_2_ batteries, and other advanced Li batteries.
